# Identification and characterization of a 25-lncRNA prognostic signature for early recurrence in hepatocellular carcinoma

**DOI:** 10.1186/s12885-021-08827-z

**Published:** 2021-10-30

**Authors:** Yi Fu, Xindong Wei, Qiuqin Han, Jiamei Le, Yujie Ma, Xinjie Lin, Yuhui Xu, Ning Liu, Xuan Wang, Xiaoni Kong, Jinyang Gu, Ying Tong, Hailong Wu

**Affiliations:** 1 Affiliated Zhoupu Hospital, Shanghai University of Medicine and Health Sciences, Shanghai, 201318 China; 2 Shanghai Key Laboratory of Molecular Imaging, Collaborative Innovation Center for Biomedicines, Shanghai University of Medicine and Health Sciences, Shanghai, 201318 China; 3 School of Medical Instruments, Shanghai University of Medicine and Health Sciences, Shanghai, 201318 China; 4 Nanjing University of Traditional Chinese Medicine, Nanjing, 210000 China; 5 Graduate School of Art and Sciences, Columbia University, New York, NY 10027 USA; 6 Department of Clinical Oncology, Taian City Central Hospital, Taian, 271000 Shandong China; 7 Department of General Surgery, Nanjing General Hospital of Nanjing Military Command, Nanjing, 210000 China; 8 Institute of Clinical Immunology, Department of Liver Diseases, Central Laboratory, Shuguang Hospital Affiliated to Shanghai University of Traditional Chinese Medicine, Shanghai, 200021 China; 9 Department of Transplantation, Xinhua Hospital Affiliated to Shanghai Jiao Tong University School of Medicine, Shanghai, 200092 China; 10 Department of Liver Surgery, Renji Hospital Affiliated to Shanghai Jiao Tong University School of Medicine, Shanghai, 200127 China

**Keywords:** Long non-coding RNA signature, Hepatocellular carcinoma, Early recurrence, Tumor infiltrating lymphocytes

## Abstract

**Background:**

Early recurrence is the major cause of poor prognosis in hepatocellular carcinoma (HCC). Long non-coding RNAs (lncRNAs) are deeply involved in HCC prognosis. In this study, we aimed to establish a prognostic lncRNA signature for HCC early recurrence.

**Methods:**

The lncRNA expression profile and corresponding clinical data were retrieved from total 299 HCC patients in TCGA database. LncRNA candidates correlated to early recurrence were selected by differentially expressed gene (DEG), univariate Cox regression and least absolute shrinkage and selection operator (LASSO) regression analyses. A 25-lncRNA prognostic signature was constructed according to receiver operating characteristic curve (ROC). Kaplan-Meier and multivariate Cox regression analyses were used to evaluate the performance of this signature. ROC and nomogram were used to evaluate the integrated models based on this signature with other independent clinical risk factors. Gene set enrichment analysis (GSEA) was used to reveal enriched gene sets in the high-risk group. Tumor infiltrating lymphocytes (TIL*s*) levels were analyzed with single sample Gene Set Enrichment Analysis (ssGSEA). Immune therapy response prediction was performed with TIDE and SubMap. Chemotherapeutic response prediction was conducted by using Genomics of Drug Sensitivity in Cancer (GDSC) pharmacogenomics database.

**Results:**

Compared to low-risk group, patients in high-risk group showed reduced disease-free survival (DFS) in the training (*p* < 0.0001) and validation cohort (*p* = 0.0132). The 25-lncRNA signature, AFP, TNM and vascular invasion could serve as independent risk factors for HCC early recurrence. Among them, the 25-lncRNA signature had the best predictive performance, and combination of those four risk factors further improves the prognostic potential. Moreover, GSEA showed significant enrichment of “E2F TARGETS”, “G2M CHECKPOINT”, “MYC TARGETS V1” and “DNA REPAIR” pathways in the high-risk group. In addition, increased TIL*s* were observed in the low-risk group compared to the high-risk group. The 25-lncRNA signature negatively associates with the levels of some types of antitumor immune cells. Immunotherapies and chemotherapies prediction revealed differential responses to PD-1 inhibitor and several chemotherapeutic drugs in the low- and high-risk group.

**Conclusions:**

Our study proposed a 25-lncRNA prognostic signature for predicting HCC early recurrence, which may guide postoperative treatment and recurrence surveillance in HCC patients.

**Supplementary Information:**

The online version contains supplementary material available at 10.1186/s12885-021-08827-z.

## Background

The very recent epidemiologic study has shown that liver cancer ranks the sixth commonly diagnosed cancer and the fourth leading cause of cancer death in the world. An estimated 84,100 liver cancer cases occurred and 78,200 liver cancer cases died in 2018 [1]. Hepatocellular carcinoma (HCC) compromises 75–85% of primary liver cancer [1]. The main clinical curative treatments for HCC include liver transplantation, percutaneous radiofrequency ablation and liver resection, among which liver resection is the most employed treatment [2]. Although 5-year overall survival rate reaches up to 50%, recurrence occurs in more than 70% HCC patients after curative surgery [3]. Clinically, the recurrence within 2-year after resection is defined as early recurrence, whereas the recurrence > 2-year is defined as late recurrence. Compared to late recurrence, HCC patients with early recurrence usually showed poorer prognosis [4].

Currently, many approaches, such as the TNM staging system of the American Joint Committee on Cancer (AJCC), the Barcelona Clinic Liver Cancer (BCLC) classification, and the Cancer of the Liver Italian Program (CLIP) staging system, have been employed to evaluate the prognosis of HCC patients [5]. However, their assessment criteria mainly rely on the clinicopathological features of HCC patients but do not take into account the critical and complicated molecular pathogenesis, an important factor in determining the outcome of HCC. Therefore, their prognostic predictive performance was unsatisfactory [6]. Meanwhile, serum alpha-fetoprotein (AFP) detection and medical imaging techniques are clinically used for post-surgery surveillance of recurrence in HCC patients, but with limited effectiveness due to the low specificity and sensitivity of those surveillance means [7].

The advent of high throughput array/sequencing and high-efficiency big data analysis in past decades makes it possible and reliable to construct multi-gene signatures to evaluate prognosis and predict therapeutic response in cancer patients. For example, a 70-gene signature had been established to aid decision making of adjuvant chemotherapy in patients with estrogen receptor-positive early breast cancer [8, 9]. More importantly, this 70-gene-signature based diagnostic test known as “MammaPrint” (Agendia, Amsterdam, The Netherlands) has been approved by the Food and Drug Administration (FDA) to predict breast cancer recurrence [10], and been validated in several retrospective studies [11, 12]. Additionally, an 18-gene signature ColoPrint (Agendia, Amsterdam, The Netherlands) was developed to predict disease relapse in patients with early-stage colorectal cancer (CRC) [13], and had been validated in other independent studies [14, 15]. Several multi-gene signatures have been constructed in HCC for prognosis evaluation. For example, Wei et al. developed a 20-miRNA signature to predict post-surgery survival in HCC patients [16]; Nault et al. constructed a 5-gene signature to evaluate the overall survival in HCC patients [17]; Kim et al. established a 233-gene signature to predict late recurrence in HCC patients [18]. Recently, prognostic signatures based on specific groups of genes such as glycolyis-related genes, metabolic-related genes and autophagy-related genes were also reported [19–21]. However, those multi-gene signatures of HCC mainly focus on overall survival and later recurrence, and few multi-gene signatures have been established to predict early recurrence in HCCs.

Long non-coding RNAs (lncRNAs) are a class of transcripts that are longer than 200 nucleotides (nt) and do not encode proteins [22]. Accumulating evidence has indicated the involvement of lncRNAs in diverse biological processes and disease pathogenesis [23]. Moreover, some lncRNAs have been reported to contribute to the initiation and progression in HCCs. For example, lncRNA-ANRIL has been reported to promote hepatocarcinoma cell proliferation [24]; and lncRNA-MALAT1 could function as a proto-oncogene to transform hepatocytes and enhance hepatocarcinoma cell growth [25]. In addition, some lncRNAs have been demonstrated to associate with HCC prognosis. For example, the overexpression of lncRNA-MVIH was associated with poor recurrence-free survival and overall survival in HCC patients [26]; LncRNA-PTTG3P expression was positively associated with tumor size, TNM stage and poor survival in HCC patients [27]. Although lncRNAs are involved in the progression and associated with prognosis in HCCs, lncRNA-based gene signatures for HCC prognostic evaluation, especially for early recurrence, are limited.

In this study, we analyzed the expression profile of lncRNAs and their association with early recurrence in the Liver Hepatocellular Carcinoma (LIHC) project from The Cancer Genome Atlas (TCGA) database (TCGA-LIHC). We constructed a 25-lncRNA signature significantly associated with HCC early recurrence. Based on this multi-lncRNA signature, HCC patients can be classified into low- and high-risk groups according to their risk scores. The early recurrence rate was significantly higher in the high-risk group than in the low-risk group. Moreover, the risk score negatively correlated with recurrence-free survival in HCC patients. Multivariate Cox regression analysis demonstrated that the 25-lncRNA signature, serum AFP, TNM stage and vascular invasion were 4 independent risk factors of HCC early recurrence. Compared with the other 3 risk factors, the 25-lncRNA signature had the best predictive performance for HCC early recurrence. Furthermore, the 25-lncRNA signature could synergize with serum AFP, TNM stage and vascular invasion to improve the prognosis evaluation for HCC early recurrence. In addition, in the context of this 25-lncRNA risk signature, we demonstrated that the “E2F TARGETS”, “G2M CHECKPOINT”, “MYC TARGETS V1” and “DNA REPAIR” were the most significantly enriched gene sets in the high-risk group. Moreover, the low-risk group showed greater tumor-infiltrating lymphocytes (TILs) compared to the high-risk group, and the 25-lncRNA prognostic signature was significantly negatively associated with the potent antitumor immune cells (i.e. type 1 T helper cell, effector memory CD8 T cell and activated CD8 T cell). Finally, the low-risk group was predicted to be more sensitive to immunotherapy like anti-PD-1 and chemotherapies like docetaxel, gefitinib and vinblastine, while the high-risk group was predicted to be more sensitive to doxorubicin, mitomycin C and paclitaxel. In conclusion, our findings may provide some insight into lncRNA-based personalized treatment and improve the strategy of post-surgery recurrence surveillance in HCC patients.

## Methods

### TCGA-LIHC database preparation and lncRNA profile mining

Gene expression profile of HCC and corresponding clinical information were downloaded from TCGA-LIHC (http://cancergenome.nih.gov/). Total 314 out of all 374 HCC samples with complete follow-up information (overall survival (OS) time, OS status, disease free survival (DFS) time and status) were retained. Among these 314 patients, some patients’ follow-up time was less than 1 month, and their OS and DFS status were labeled as “alive” and “recurrence free”. Therefore, these patients were not suitable for early recurrence analysis and they were excluded. Thus, we used 299 patients for signature construction in this study. The 299 HCC patients were then randomly divided into a training cohort (*N* = 150) and a validation cohort (*N* = 149). Based on the information of annotated lncRNAs in GENCODE V30, 14,847 human lncRNAs with Ensembl gene ID were obtained and their corresponding expression profile was extracted from the TCGA-LIHC.

### Construction and validation of lncRNA-based risk signature

Most bioinformatics analyses were conducted using R software. DEG analysis was performed between the 150 HCC samples in the training cohort and 50 normal tissue samples from TCGA-LIHC project by using R package “edgeR” [28, 29]. Univariate Cox regression analysis was performed to select early recurrence related lncRNAs by using R package “survival” [30]. FunRich (version 3) was used to draw Venn diagram between differentially expressed lncRNAs and early recurrence related lncRNAs to obtain candidate lncRNAs for signature construction [31]. Candidate lncRNAs were then further analyzed in LASSO regression analysis by running R package “glmnet” for 1000 times [32], and the most powerful prognostic lncRNAs were selected through 10-fold cross-validation with lambda.min as the optimized cut-off [33]. Risk score of each patient was calculated in a linear combination of lncRNAs weighted by their corresponding regression coefficients and expression levels in indicated HCC patients by formula (*risk score* =  ∑ *coefficient* × *expression*(*gene*)). Receiver operating characteristic curve (ROC) analysis was conducted by using R package “pROC” [34], and the predictive performance was assessed by calculating the area under curve (AUC). Finally, a combination of 25 lncRNAs was chosen for establishing risk signature because this 25-lcnRNA risk signature gave the largest AUC in ROC analysis. The 150 HCC patients were divided into the low-risk group (*N* = 75) and the high-risk group (*N* = 75) by using the median risk score as cut-off. A correlation analysis was performed between the risk score and early recurrence. Kaplan-Meier analysis, cumulative hazard and cumulative events analyses were conducted by using R package “survival” in the training cohort, the validation cohort and the total 299 HCC patients to investigate the early recurrence survival between low risk patients and high risk patients. Univariate and multivariate Cox analysis were done in the total 299 HCC patients with R package “survival” to evaluate whether the risk score could serve as an independent factor for early recurrence prediction in HCCs. Nomogram was constructed by using the 25-lncRNA signature, AFP, vascular invasion, TNM stages and their corresponding multivariate Cox regression coefficients, and calibration plots were generated with R package “regplot” [35]. C-index was used to evaluate the model performance for predicting early recurrence.

### Gene set enrichment analysis (GSEA)

GSEA was conducted by using GSEA JAVA program (version 4.0.3) downloaded from official website (http://software.broadinstitute.org/gsea/index.jsp) to find out enriched gene sets. MsigDB h.all.v7.1.symbols.gmt gene set collection was chosen for identifying hallmarks of HCC early recurrence. The random sample permutations were set to be 1000 with the significance set as |NES| > 1, FDR q < 0.25 and nominal *P* < 0.05.

### Analysis of the levels of tumor-infiltrating lymphocytes and immune therapy response prediction

Immune infiltration analysis was performed with single sample Gene Set Enrichment Analysis (ssGSEA) by using “GSVA” package in R [36]. A group of 28 cellmarker sets were used for calculating normalized enrichment score (NES) for each cell type in every 299 HCC samples [37]. Correlation analysis between risk scores and NES of immune cells was performed by function “cor.test” in R. TIDE (Tumor Immune Dysfunction and Exclusion) algorithm and SubMap modules from GenePattern were used to predict the response to immune checkpoint blockade for all 299 HCC samples [38–40].

### Analysis of chemotherapeutic response prediction

Chemotherapeutic response prediction for every 299 HCC samples was conducted in R by using “pRRophetic” package based on the Genomics of Drug Sensitivity in Cancer (GDSC) pharmacogenomics database. The half maximal inhibitory concentration (IC_50_) was estimated by ridge regression and the prediction accuracy was evaluated by 10-fold cross-validation [41].

### Real time quantitative RT-PCR

To validate the 25-lncRNA signature in clinical samples, 3 lncRNAs from the signature were selected and their relative expressions in HCC samples were detected by RT-qPCR. Total RNA from 36 paired HCC tumor and adjacent tissues provided by Xinhua Hospital were extracted by using TRIzol (Invitrogen, 15596026) according to the manufacturer’s instructions. cDNA was synthesized by using ReverTra Ace® qPCR RT Master Mix with gDNA Remover (TOYOBO, FSQ-301) in a SimpliAmp Thermal Cycler (Applied Biosystems). The 20 μL PCR reaction system consist of 2 μL cDNA, 0.8 μL forward primer, 0.8 μL reverse primer, 10 μL CYBR *Premix Ex Taq*II, 0.4 μL ROX Reference Dye II and 6 μL deionized water (Takara CYBR *Premix Ex Taq*II, RR820A). RT-PCR was performed in ABI Biosystems™ 7500 Real-Time qPCR System (Applied Biosystems). 18s was used as a housekeeping gene for normalization and the relative expression of selected genes was calculated by using 2^−ΔΔCT^ method. Primers used were synthesized by GENEWIZ and the sequences of primers were ENSG00000231918 (GTGGCTCTGCCTTGGGTAAT, TTCCAGAACAACCTTGTCAGA), ENSG00000248596 (GCCAGAATTGGCGGTTTCTC, ATCGCTGAGTGTGTCGAGTG), and ENSG00000223392 (ATCCTTACCCTGCATTGCCC, ATGATCCAACCATCTGCAGGG).

### Statistical analysis

DeLong’s test was used to compare the sensitivity and specificity of two ROC curves. Chi-square test was used to evaluate the impact of risk score group distribution on recurrence cases between 1 year and 2 years. The correlation of risk scores with disease free survival (DFS), NES of tumor-infiltrating lymphocytes and levels of immune checkpoints was analyzed by nonparametric Spearman’s rank correlation analysis. The log-rank test was used for Kaplan-Meier survival analyses, cumulative hazard and cumulative events analyses. The Cox proportional hazards regression model was used for univariate and multivariate analyses. Wilcoxon test was used for comparing NES of immune cells and IC_50_ of drugs between the low-risk and high-risk group. The difference was considered statistically significant when *P* < 0.05 in all statistical analysis.

## Results

### HCC dataset preparation and identification of candidate lncRNAs from the training cohort

**Fig. 1:**
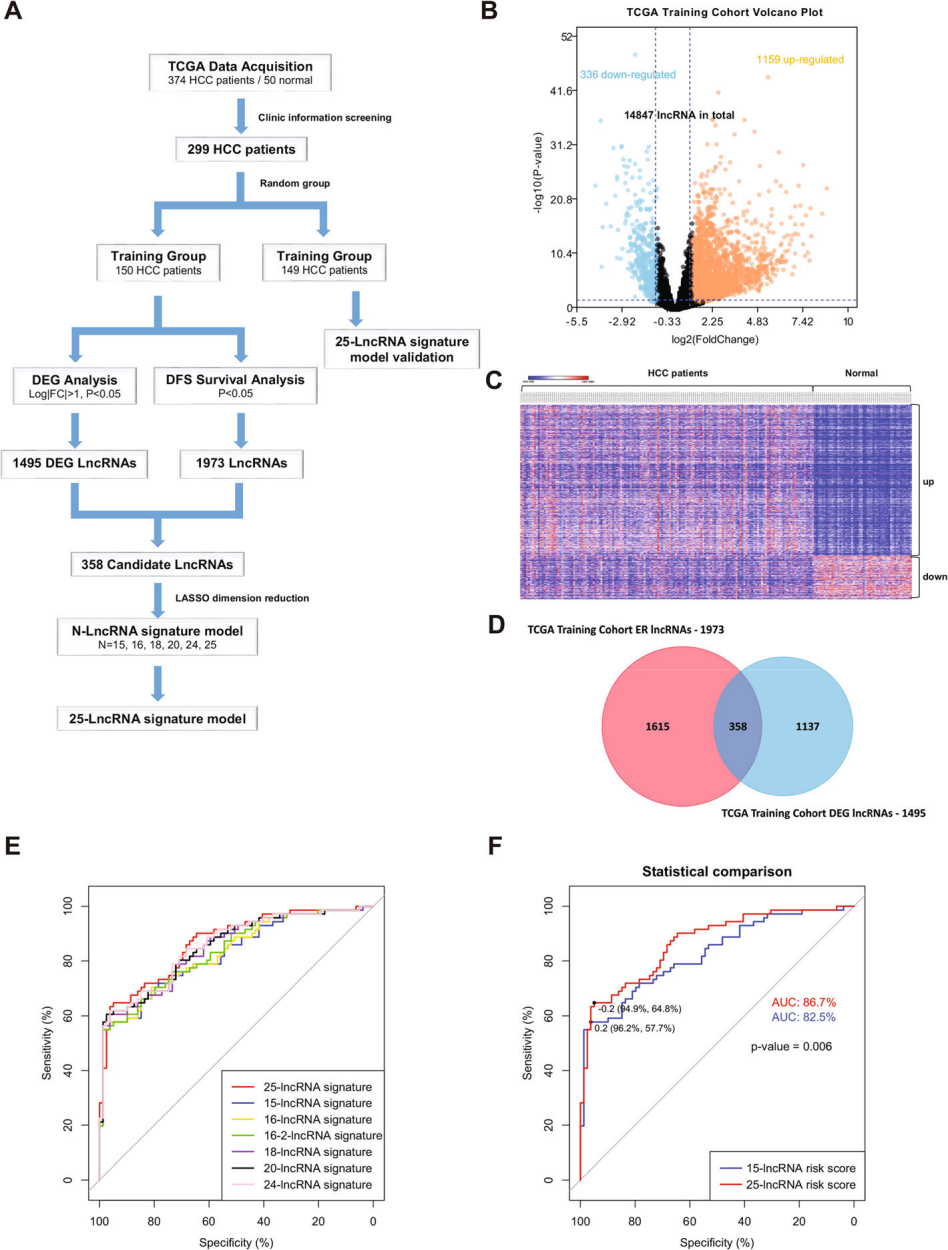
Data processing and lncRNA-based early risk signature construction from candidate lncRNAs. **A**) Schematic diagram of data processing and construction of lncRNA-based signature; **B**) Volcano plot of lncRNAs expression in the TCGA training cohort. Differentially expressed gene (DEG) analysis shows 1159 up-regulated lncRNAs and 336 down-regulated lncRNAs; **C**) Heatmap of 1495 DEG lncRNAs in 150 HCC samples and 50 normal tissues; **D**) Venn plot of DEG lncRNAs and early recurrence related lncRNAs (ER lncRNAs) in the TCGA training cohort. 358 DEG lncRNAs with potential prognostic value for HCC early recurrence were identified; **E**) ROC plot of 7 lncRNA signatures; **F**) ROC plot comparison between the 15-lncRNA risk signature and the 25-lncRNA risk signature, *P* = 0.006

HCC RNA-seq data and corresponding clinical information were downloaded from the TCGA-LIHC (Liver Hepatocellular Carcinoma) project. After removal of samples without complete survival information, total 299 out of all 374 HCC samples were enrolled in this study for further analysis. Table S1 shows the clinical characteristics of the 299 HCC samples, in which more than 50% HCC patients had recurrence. Because there are no suitable GEO datasets which are comparable to the TCGA-LIHC project containing comprehensive data on both lncRNA expression profile and patients’ clinical characteristics, we then randomly divided the 299 HCC patients into a training cohort (*n* = 150) and a validation cohort (*n* = 149) by using “split” function in R software instead of setting an external validation cohort. Bioinformatics analyses were first performed in the training cohort and further validated in the validation cohort (Fig. 1A).

To establish a lncRNA-based risk signature, differentially expressed gene (DEG) analysis on lncRNAs was performed between the training cohort and 50 normal controls from the TCGA-LIHC project. A total of 1495 DEG lncRNAs were found significantly dysregulated in HCC samples (1159 up-regulated and 336 down-regulated, log2|FC| > 1, FDR < 0.05) (Fig. 1B and C). Meanwhile, a univariate Cox regression analysis revealed that total 1973 lncRNAs were associated with HCC early recurrence (ER lncRNAs) (*P* < 0.05) (Fig. 1D). Finally, a Venn diagram between the 1495 DEG lncRNAs and the 1973 ER lncRNAs identified 358 lncRNA candidates which may have potential prognostic value for HCC early recurrence (Fig. 1D).

### Pilot construction of multi-lncRNA signatures for HCC early recurrence

The least absolute shrinkage and selection operator (LASSO) Logistic Regression is a selection and shrinkage technique designed for regression model initially applied to Ordinary Logistic Regression [42]. LASSO can better identify those risk factors strongly linked to the outcome and is widely employed in signature construction [43]. To identify key lncRNAs suitable for establishing a risk signature for predicting HCC early recurrence, those 358 candidate lncRNAs (Fig. 1D) were further analyzed in LASSO regression. A total of 1000 LASSO regression iterations were performed by using the R package “glmnet”. Lambda.min was chosen as the optimized cut-off to select key lncRNAs for risk model (Fig. S1) [44]. Consequently, 7 lncRNA combinations were obtained after LASSO analysis (Table S2 and Fig. S1). Thus, 7 lncRNA risk signatures were individually constructed based on these combinations. The risk score of each HCC patient was calculated in a linear formula *risk score* =  ∑ *coefficient* × *expression*(*gene*) (expression: lncRNA expression in individual HCC patients; coefficient: regression coefficients of indicated lncRNAs). To determine which lncRNA risk signature gives the best predictive performance on early recurrence, receiver operating characteristics (ROC) analysis was conducted between the 7 lncRNA risk signatures. As shown in Fig. 1E, all the 7 lncRNA risk signatures gave high area under the ROC curve (AUC, AUC > 80%), suggesting the reliability of our LASSO analysis. Among them, the 25-lncRNA risk signature gave the highest AUC (AUC = 86.70%) (Fig. 1F), suggesting the 25-lncRNA risk signature has the best predictive performance for HCC early recurrence.

### Risk score calculation of the 25-lncRNA risk signature

Since the 25-lncRNA risk signature gave the best predictive performance, we then selected this signature to establish a risk model for HCC early recurrence. The detailed information of the 25 lncRNAs, including Ensembl gene ID, gene symbol, hazard ratio and coefficients, was summarized in Table 1. Among them, 19 lncRNAs (ENSG00000253417, ENSG00000272205, ENSG00000269894, ENSG00000275437, ENSG00000223392, ENSG00000248596, ENSG00000268201, ENSG00000247675, ENSG00000231918, ENSG00000234129, ENSG00000269974, ENSG00000236366, ENSG00000275223, ENSG00000253406, ENSG00000232079, ENSG00000255980, ENSG00000267905, ENSG00000176912, ENSG00000254333) had positive coefficients and were negatively associated with disease free survival (DFS), and the remainder 6 lncRNAs (ENSG00000259834, ENSG00000254887, ENSG00000259974, ENSG00000273837, ENSG00000231246, ENSG00000234283) had negative coefficients and were positively associated with DFS (Table 1). Here, we named those lncRNAs with positive coefficients as risk lncRNAs and those with negative coefficients as protective lncRNAs. The risk score could be calculated according to the coefficients of individual lncRNAs and their expression in corresponding HCC patients.

**Table 1:** LncRNAs significantly associated with the disease free survival in the training cohort patients (*N* = 150)

Ensembl	Gene Symbol	***P*** value ^**a**^	Hazard Ratio ^**a**^	Coefficient ^**b**^	associated diseases	Description	Reference
ENSG00000253417	LINC02159	< 0.001	2.85	0.069	colorectal cancer, melanoma, head and neck squamous cell carcinoma	long intergenic non-protein coding RNA 2159	[1–3]
ENSG00000272205		< 0.001	2.47	0.233	NR	NR	
ENSG00000269894		< 0.001	2.48	0.092	NR	NR	
ENSG00000275437		< 0.001	4.78	0.083	NR	NR	
ENSG00000223392	CLDN10-AS1	< 0.001	1.82	0.278	atherogenesis, lung adenocarcinoma, colorectal cancer, thyroid cancer, cholangiocarcinoma, colon adenocarcinoma	CLDN10 antisense RNA 1	[4–6, 20]
ENSG00000248596	LOC643201	< 0.001	1.91	0.335	colorectal cancer	centrosomal protein 192 kDa pseudogene	[5]
ENSG00000268201		< 0.001	1.98	0.330	NR	NR	
ENSG00000247675	LRP4-AS1	< 0.001	7.36	0.567	breast cancer, pancreatic neuroendocrine tumour	LRP4 antisense RNA 1	[7]
ENSG00000231918	LOC730100	< 0.001	2.19	0.339	glioma	uncharacterized LOC730100	[8]
ENSG00000259834		< 0.001	0.28	−0.721	NR	NR	
ENSG00000234129		0.001	9.68	0.272	NR	NR	
ENSG00000269974		0.001	2.24	0.007	NR	NR	
ENSG00000254887	LOC100505622	0.002	0.05	−0.735	gastric cancer	uncharacterized LOC100505622	[12]
ENSG00000259974	LINC00261	0.003	0.69	− 0.187	hepatocellular carcinoma, endometrial carcinoma, non-small cell lung cancer, colon cancer, esiohageal cancer, endometriosis, choriocarcinoma, gastric cancer, esophageal cancer, lung epithelial homeostasis, endoderm differentiation	long intergenic non-protein coding RNA 261	[13–19, 25–27]
ENSG00000236366	LOC153910	0.003	1.65	0.232	lung function development, chronic obstructive pulmonary disease (COPD) and cardiovascular diseases (CVD)	uncharacterized LOC153910	[3, 21–24]
ENSG00000275223		0.003	1.56	0.024	NR	NR	
ENSG00000253406		0.004	967.38	1.878	NR	NR	
ENSG00000232079	LINC01697	0.009	1.53	0.142	lung squamous cell carcinoma, gastric cancer, gastric adenocarcinoma, breast cancer	long intergenic non-protein coding RNA 1697	[9–11]
ENSG00000273837		0.012	0.59	−0.025	NR	NR	
ENSG00000255980	LOC102724265	0.017	10.02	0.125	NR	uncharacterized LOC102724265	
ENSG00000267905		0.018	1.5	0.032	NR	NR	
ENSG00000176912	TYMSOS	0.023	1.28	0.038	NR	TYMS opposite strand	
ENSG00000254333	NDST1-AS1	0.032	1.21	0.044	NR	NDST1 antisense RNA 1	
ENSG00000231246	LINC02884	0.033	0.17	−0.041	NR	long intergenic non-protein coding RNA 2884	
ENSG00000234283		0.047	0.39	−0.459	NR	NR	

*NR* not reported

^a^ Derived from the univariable Cox proportional hazards regression analysis in the 150 training cohort patients

^b^ Derived from the LASSO regression analysis in the 150 training cohort patients

Please refer to the supplementary material for references citation

### The 25-lncRNA risk signature correlates with HCC early recurrence

**Fig. 2:**
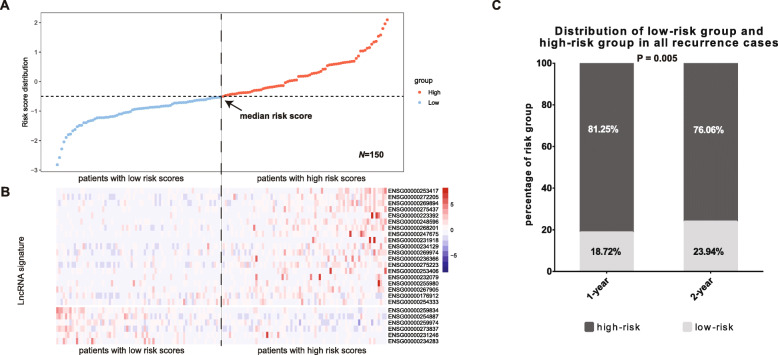
Correlation analysis of the 25-lncRNA risk signature with HCC early recurrence in the training cohort. **A**) The 150 HCC patients in the training cohort was ranked according to their risk scores from low to high, and the median risk score was set as the cut-off to divide the 150 HCC patients into low-risk group (*n* = 75) and high-risk group (*n* = 75); **B**) The 25-lncRNA expression profile in the 150 HCC patients. The 19 risk lncRNAs were enriched in the high-risk group and the 6 protective lncRNAs were enriched in the low-risk group; **C**) 81.25% and 76.06% HCC patients with recurrence in 1-year and 2-year respectively were classified in the high-risk group, and 18.72% and 23.94% HCC patients with recurrence in 1-year and 2-year respectively were classified in the low-risk group (*P* = 0.005)

To determine whether the 25-lncRNA risk signature could predict HCC early recurrence, we first calculated the risk scores of the 150 HCC patients in the training cohort and then distributed them according to their risk scores from low to high (Fig. 2A). The median risk score was set as the cut-off to separate those patients into low-risk group (*n* = 75, patients’ risk scores < the median risk score) and high-risk group (*n* = 75, patients’ risk scores > the median risk score) (Fig. 2A). As shown in Fig. 2B, the 19 risk lncRNAs were mostly enriched in the high-risk group whereas the 6 protective lncRNAs were mainly enriched in the low-risk group. Moreover, 81.25% of recurrence cases in 1-year and 76.06% in 2-year came from the high-risk group, while the percentages were respectively 18.75 and 23.94% in the low-risk group (Fig. 2C). These results indicate that the 25-lncRNA risk signature have satisfying predictive potential for HCC early recurrence in the training cohort.

### Validation of the 25-lncRNA risk signature

**Fig. 3:**
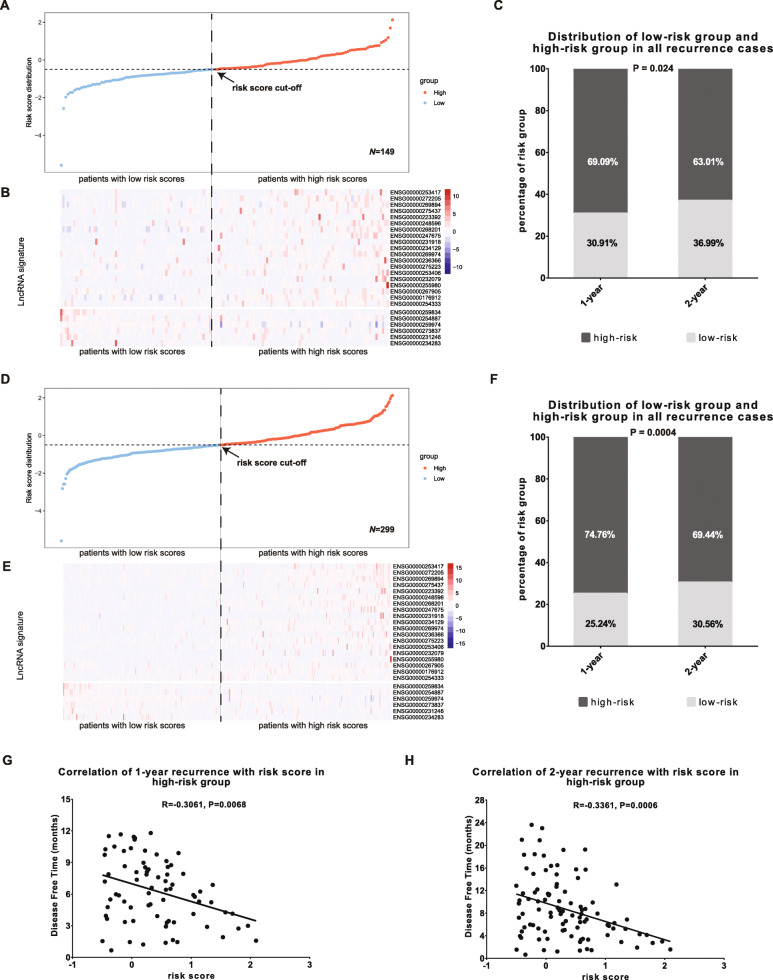
Correlation analysis of the 25-lncRNA risk signature with HCC early recurrence in the validation and entire TCGA cohort. **A**) The 149 HCC patients in the validation cohort were ranked according to their risk scores from low to high, and divided into the low-risk group (*n* = 69) and the high-risk group (*n* = 80) by using the same risk score cut-off in the training cohort; **B**) The 25-lncRNA expression profile in the 149 HCC patients. The 19 risk lncRNAs were enriched in the high-risk group and the 6 protective lncRNAs were enriched in the low-risk group; **C**) 69.09% and 63.01% HCC patients with recurrence in 1-year and 2-year respectively were classified in the high-risk group, and 30.91% and 36.99% HCC patients with recurrence in 1-year and 2-year respectively were assigned in the low-risk group (*P* = 0.024); **D**) The 299 HCC patients in the entire TCGA cohort were ranked according to their risk scores from low to high, and divided into the low-risk group (*n* = 144) and the high-risk group (*n* = 155) by using the same risk score cut-off in the training cohort; **E**) The 25-lncRNA expression profile in the 299 HCC patients. The 19 risk lncRNAs were enriched in the high-risk group and the 6 protective lncRNAs were enriched in the low-risk group; **F**) 74.46% and 69.44% HCC patients with recurrence in 1-year and 2-year respectively were assigned in the high-risk group, and 25.24% and 30.56% HCC patients with recurrence in 1-year and 2-year respectively were assigned in the low-risk group (*P* = 0.0004); G and H) Correlation of risk score with 1-year (**G**) or 2-year DFS (**H**) in the recurrent HCC patients in the high-risk group of the entire TCGA cohort

To validate the predictive potential of the 25-lncRNA risk signature, we evaluated it in the validation cohort. According to the median cut-off in the training cohort, the validation cohort (*n* = 149) was separated into the low-risk group (*n* = 69) and the high-risk group (*n* = 80) (Fig. 3A). In line with the finding in the training cohort, the risk lncRNAs were mainly enriched in the high-risk group whereas the protective ones were mainly enriched in the low-risk group (Fig. 3B). Meanwhile, 69.09% of 1-year recurrence cases and 63.01% of 2-year recurrence cases came from the high-risk group (Fig. 3C). Moreover, the predictive potential of the 25-lncRNA risk signature was also evaluated in the total 299 recruited HCC patients. Similarly, 299 HCC patients were separated into low-risk group (*n* = 144) and high-risk group (*n* = 155) according to the median cut-off in the training cohort (Fig. 3D). Most of the risk lncRNAs were enriched in the high-risk group and most of the protective lncRNAs were enriched in the low-risk group (Fig. 3E). Consistent with this finding, non-pair Wilcoxon test confirmed enrichment of the risk lncRNAs and the protective lncRNAs in the high-risk and low-risk groups respectively, except for the lncRNAs (ENSG00000255980, ENSG00000253406, ENSG00000232079, and ENSG00000234283) whose expression showed no significant changes between the low- and high-risk groups (Fig. S2). Patients in the high-risk group contributed 74.76% of 1-year recurrence cases and 69.44% of 2-year recurrence cases (Fig. 3F). More importantly, correlation assays showed significantly negative correlation of risk score with 1-year (Fig. 3G) or 2-year DFS (Fig. 3H) in the recurrent HCC patients in the high-risk group. No correlation was observed between risk score and DFS in recurrent HCC patients in the low-risk group (Fig. S3). These findings further validate the correlation of the 25-lncRNA risk signature with HCC early recurrence and indicate the great predictive potential of the risk signature on HCC early recurrence.

The primary purpose for the signature construction study is to accurately discriminate low- and high-risk patients. Therefore, the cut-off selection is critical for the accuracy of the prediction signature. In this study, we adopted the median risk score as cut-off which has been widely employed by many other groups [16, 45–48]. To investigate whether there are other cut-offs which could distinguish low- and high-risk of early recurrence better than the median cut-off, we adopted the cut-off derived from Youden index [49]. Although the Youden index/cut-off could separate patients into the low- and high-risk groups (Fig. S4A-C), the prediction performance for early recurrence is much poorer than that by using median cut-off (Fig. S4D-F). Therefore, the median risk score used in this study is an appropriate cut-off to accurately distinguish HCC patients with low or high early recurrence risk.

### The 25-lncRNA risk signature precisely predicts early recurrence in HCC patients

**Fig. 4:**
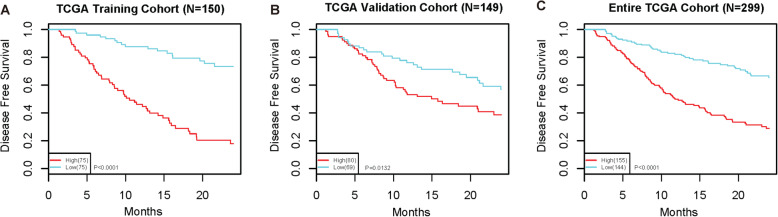
Kaplan-Meier analysis of the association of the 25-lncRNA risk signature with early recurrence risk in HCCs. **A**-**C**) The association of the 25-lncRNA risk signature with 2-year DFS was analyzed in the training cohort (*N* = 150, *P* < 0.0001) (**A**), validation cohort (*N* = 149, *P* = 0.0132) (**B**), and the entire TCGA cohort (*N* = 299, *P* < 0.0001) (**C**). The statistical significance was determined by the log-rank test. The patients in each cohort were stratified into the high-risk and low risk groups based on the cut-off risk score in the training cohort

To further investigate the prognostic value of the 25-lncRNA risk signature for early recurrence, we analyzed cumulative hazard and event in HCC patients. Both cumulative hazards and cumulative events were significantly higher in the high-risk group than those in the low-risk group in either the training cohort (Fig. S5A and B), validation cohort (Fig. S5C and D) or total 299 HCC patients (Fig. S5E and F). Meanwhile, Kaplan-Meier analyses, in the training cohort (Fig. 4A), validation cohort (Fig. 4B) and 299 enrolled HCC patients (Fig. 4C), showed that the patients in the high-risk group had lower 2-year DFS than those in the low-risk group. These findings further indicate the prognostic value of the 25-lncRNA risk signature for HCC early recurrence.

### The 25-lncRNA risk signature is an independent prognostic factor for early recurrence in HCCs

To determine whether the 25-lncRNA risk signature is an independent prognostic factor for HCC early recurrence, we performed univariate and multivariate Cox regression analyses in the enrolled 299 HCC patients. The 25-lncRNA risk score and other clinicopathological factors, including gender, age, race, cirrhosis, vascular invasion, serum AFP level and TNM stage, were used as covariates. As shown in Table 2, the vascular invasion, serum AFP and 25-lncRNA risk score were significantly associated with 1-year and 2-year recurrence in HCC patients, while the TNM stage was significantly associated with 2-year recurrence but not with 1-year recurrence. These findings are consistent with previous studies showing that serum AFP [50], TNM stage [51] and vascular invasion [2] are independent risk factors for HCC early recurrence, and indicate that the 25-lncRNA risk signature could serve as an independent prognostic factor for HCC early recurrence.

**Table 2:** Univariate and multivariate Cox analysis of risk factors in the TCGA entire group (*N* = 299)

Characteristics	Univariate			Multivariate		
	Hazard Ratio	CI 95	***P*** Value	Hazard Ratio	CI 95	***P*** Value
**1-year DFS**						
risk score of 25-lncRNA signature	2.38	1.88–3.01	< 0.001	2	1.48–2.71	< 0.001
TNM Stages	1.96	1.55–2.47	< 0.001	1.28	0.94–1.75	0.124
Vascular Invasion	1.88	1.35–2.61	< 0.001	1.66	1.12–2.45	0.011
AFP	2.52	1.56–4.07	< 0.001	2.23	1.34–3.74	0.002
Cirrhosis	1.11	0.65–1.9	0.709			
Gender	1.17	0.78–1.77	0.444			
Age	0.77	0.49–1.22	0.269			
Race	1.15	0.83–1.59	0.396			
**2-year DFS**						
risk score of 25-lncRNA signature	2.19	1.8–2.66	< 0.001	1.92	1.48–2.49	< 0.001
TNM Stages	1.96	1.61–2.39	< 0.001	1.46	1.12–1.89	0.005
Vascular Invasion	1.8	1.36–2.38	< 0.001	1.5	1.05–2.12	0.024
AFP	2.03	1.37–2.99	< 0.001	1.77	1.16–2.71	0.008
Cirrhosis	1.25	0.81–1.93	0.305			
Gender	1.21	0.86–1.71	0.27			
Age	0.91	0.6–1.36	0.635			
Race	0.84	0.62–1.12	0.229			

In univariate and multivariate Cox analysis, risk score, TNM stages and vascular invasion were evaluated as continuous variables, and AFP, cirrhosis, gender, age and race were evaluated as category variables. Age category and AFP category were defined by 50 and 20 ng/ml as cut-off value, respectively

### The combination of the 25-lncRNA risk signature, AFP, TNM stage and vascular invasion improves the prognosis evaluation and the construction of nomogram

**Fig. 5:**
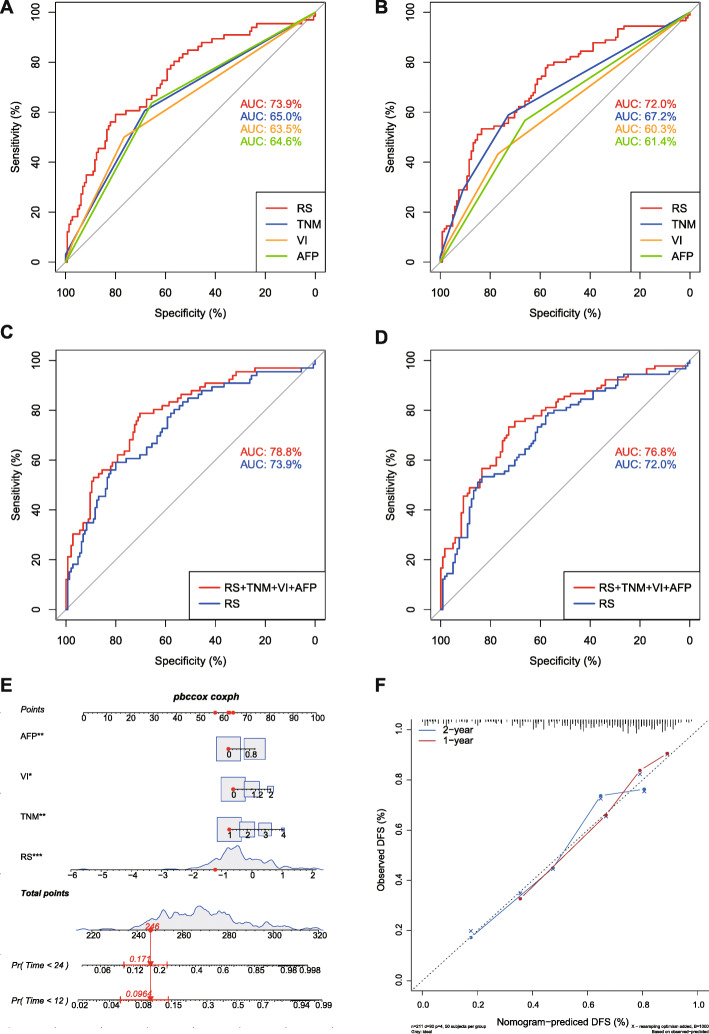
ROC analysis of the predictive performance and nomogram construction for early recurrence of the 25-lncRNA risk signature, TNM stage, vascular invasion and AFP. **A**-**B**) ROC analysis of the predictive performance of the 25-lncRNA risk signature, TNM stages, vascular invasion and AFP for 1-year DFS (**A**) and 2-year DFS (**B**) in the entire TCGA cohort; **C**-**D**) ROC analysis of the predictive performance of the combination of the 25-lncRNA risk signature, TNM stages, vascular invasion and AFP and risk score alone for 1-year DFS (**C**) and 2-year DFS (**D**) in the entire TCGA cohort. **E**) Nomogram of the 25-lncRNA signature risk score combined with AFP, vascular invasion and TNM stages; **F**) Calibration curves for the 25-lncRNA-signature-integrated nomogram for 1-year DFS and 2-year DFS. RS: the risk score of the 25-lncRNA signature, VI: vascular invasion

To investigate which independent risk factor gives the best predictive performance for HCC early recurrence, ROC analyses were performed by using “pROC”. As shown in Fig. 5A and B, the AUC of risk score for 1-year recurrence (73.86%) and 2-year recurrence (71.98%) were better those of AFP (64.58% for 1-year, 61.39% for 2-year recurrence), TNM (64.99% for 1-year, 67.17% for 2-year recurrence) and vascular invasion (VI) (63.47% for 1-year, 60.33% for 2-year recurrence). Moreover, compared to risk score alone, combining the risk score with AFP, TNM and VI further increased the predictive performance for 1-year recurrence (AUC: 78.79% vs. 73.86%) and 2-year recurrence (AUC: 76.82% vs. 71.98%) (Fig. 5C and D). The 95% confidence interval of AUC and C-index of above signatures were summarized in Table S3. An integrated Nomogram was further constructed by combining the 25-lncRNA signature, AFP, VI and TNM with a C-index 0.739 (Fig. 5E), and the calibration curves of the integrated nomogram for 1-year and 2-year DFS were presented in Fig. 5F. Therefore, the combination of the 25-lncRNA risk signature with AFP, TNM and VI could improve the prognosis evaluation for HCC early recurrence.

### Biological processes associated with HCC early recurrence

**Fig. 6:**
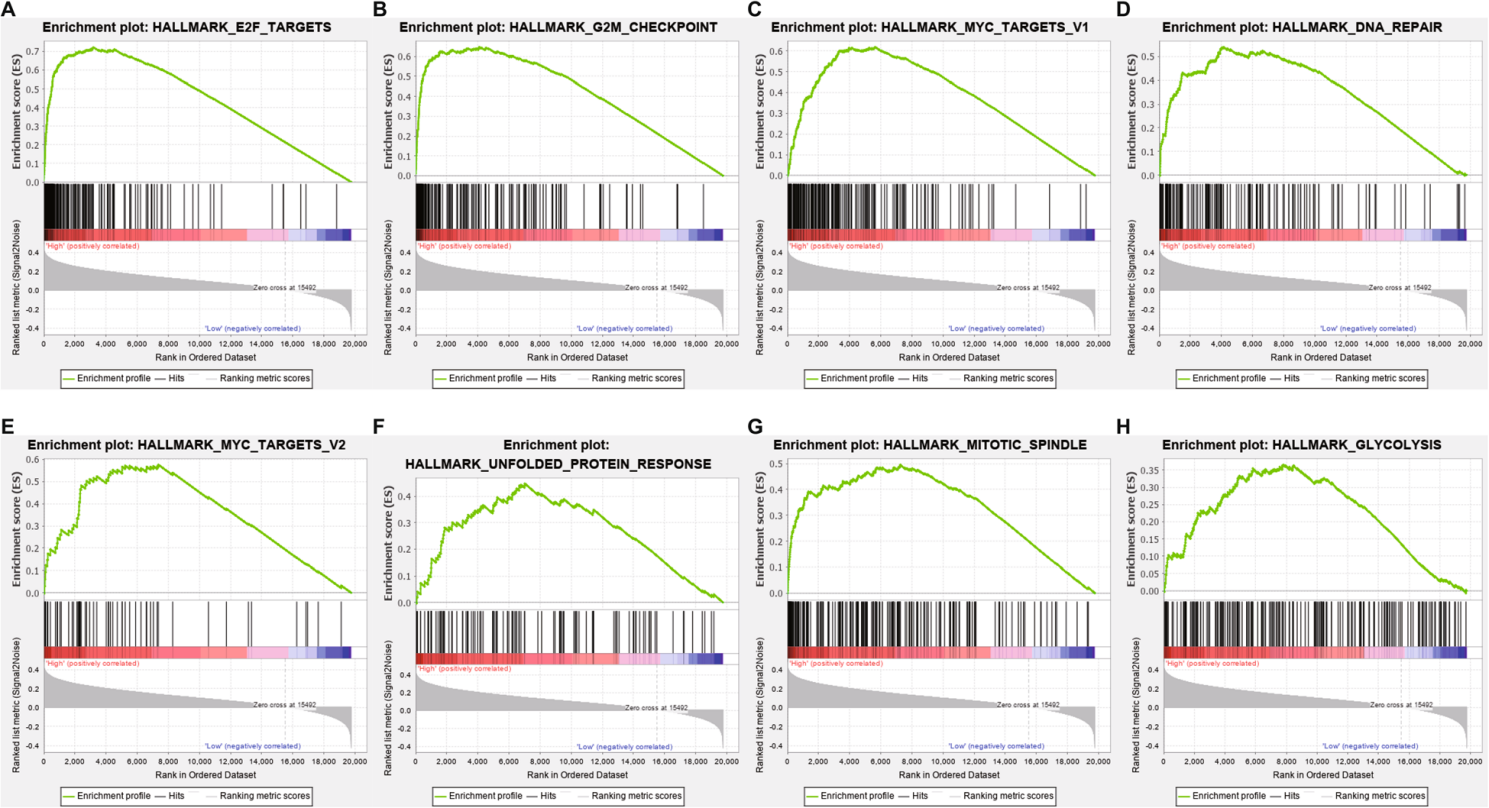
Gene set enrichment analysis illustrated upregulated gene sets in the high-risk group. **A**) Enrichment plot: HALLMARK_E2F_TARGETS; **B**) Enrichment plot: HALLMARK_G2M_CHECKPOINT; **C**) Enrichment plot: HALLMARK_MYC_TARGETS_V1; **D**) Enrichment plot: HALLMARK_DNA_REPAIR; **E**) Enrichment plot: HALLMARK_MYC_TARGETS_V2; **F**) Enrichment plot: HALLMARK_UNFOLDED_PROTEIN_RESPONSE; **G**) Enrichment plot: HALLMARK_MITOTIC_SPINDLE; **H**) Enrichment plot: HALLMARK_GLYCOLYSIS. |NES| > 1, FDR < 0.25, *P* < 0.05

Previous studies have shown that lncRNAs function as key regulators of critical biological processes including cell differentiation, development, and apoptosis [52]. To investigate the biological processes associated with HCC early recurrence, gene set enrichment analysis (GSEA) was performed with hallmark pathways based on the gene expression profiling data from HCC patients in the high-risk and the low-risk groups. Eight gene sets were significantly enriched in the high-risk group while no significant gene set enrichment was observed in the low-risk group (|NES| > 1, FDR q-val < 0.25, NOM p-val < 0.05) (Table 3). Among them, the enrichment of gene sets of “E2F TARGETS”, “G2M CHECKPOINT”, “MYC TARGETS V1” and “DNA REPAIR” showed higher significance (|NES| > 1.5, FDR q-val < 0.10, NOM p-val < 0.01) (Table 3). The snapshots of enrichment results were displayed in Fig. 6 and the heatmaps for enriched gene sets were displayed in Fig. S6. These findings suggest that the 25 lncRNAs may affect HCC early recurrence through E2F, Myc, G2M and DNA repair pathways.

**Table 3:** GSEA pathways up-regulated in high-risk group

Gene Sets	SIZE	NES	NOM p-val	FDR q-val
HALLMARK_E2F_TARGETS	195	1.991	0.000	0.045
HALLMARK_G2M_CHECKPOINT	189	1.845	0.002	0.077
HALLMARK_MYC_TARGETS_V1	194	1.844	0.004	0.052
HALLMARK_DNA_REPAIR	148	1.826	0.004	0.045
HALLMARK_MYC_TARGETS_V2	58	1.641	0.040	0.153
HALLMARK_UNFOLDED_PROTEIN_RESPONSE	107	1.562	0.043	0.207
HALLMARK_MITOTIC_SPINDLE	198	1.542	0.019	0.199
HALLMARK_GLYCOLYSIS	198	1.516	0.022	0.205

### The 25-lncRNA signature negatively associates with tumor infiltrating lymphocytes

**Fig. 7:**
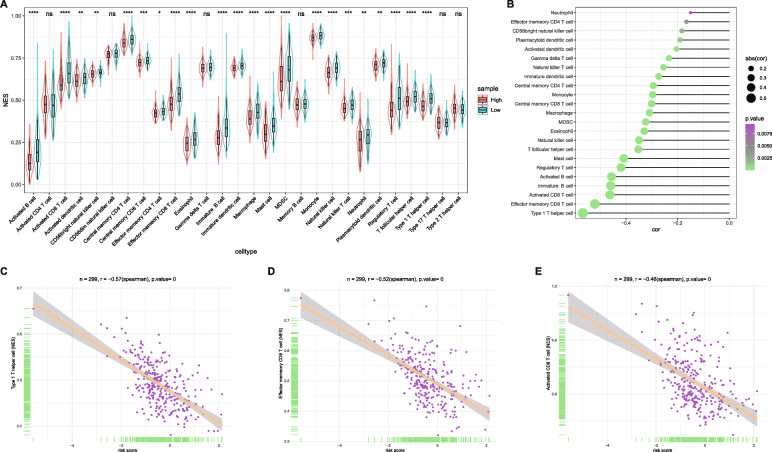
Association of the 25-lncRNA signature risk score with immune infiltration of 299 HCC samples. **A**) Comparisons of NES of immune cells between the low-risk and high-risk group, 22 immune cells showed higher NES in the low-risk group (*P* < 0.05); **B**) Correlation between risk scores and NES of immune cells, 23 immune cells were negatively associated with risk scores (*P* < 0.05); **C**)-**E**) Representative correlations between risk scores and type 1 T helper cell (**C**), effector memory CD8 T cell (**D**), activated CD8 T cell (**E**), |NES| > 0.4

Tumor infiltrating lymphocytes (TILs) have been recognized as a prognostic factor in various types of cancers, and accumulation of TILs has been established as a positive prognostic factor in a number of solid cancers including melanoma [53], colon cancer [54] and ovarian cancer [55]. Previous studies have demonstrated that HCC patients with prominent TILs showed reduced recurrence and better prognosis compared with those without prominent TILs [56, 57]. Although the TILs are minority in the tumor bulk, the immune checkpoint molecules specifically express on T cells and antigen presenting cells but not tumor cells or other stromal cells in tumor bulk. Thus, it is a commonly accepted approach to evaluate TILs by using the expression levels of immune checkpoint molecules from bulk-tumor data [45, 58, 59]. To investigate whether the 25-lncRNA prognostic signature could reflect the levels of TILs, comparison of TILs was performed between the low- and high-risk groups. As shown in Fig. 7A, 22 out of 28 TILs showed significant enrichment in the low-risk group compared to the high-risk group (*P* < 0.05). Correlation analysis between risk scores and normalized enrichment scores (NES) of TILs revealed that the intratumor accumulation of 23 TILs was negatively associated with risk scores (*P* < 0.05, Fig. 7B). Among them, the type 1 T helper cell, effector memory CD8 T cell and activated CD8 T cell, which are well-known antitumor immune cells, ranked as the top 3 TILs negatively associated with the risk scores (|NES| > 0.4, Fig. 7C-E). These findings suggested that the 25-lncRNA prognostic signature may reflect the levels of TILs and predict the post-surgery prognosis in HCCs.

### The low-risk group patients showed more sensitivity in immunotherapies and the low- and high-risk group patients showed different chemotherapies responses

**Fig. 8:**
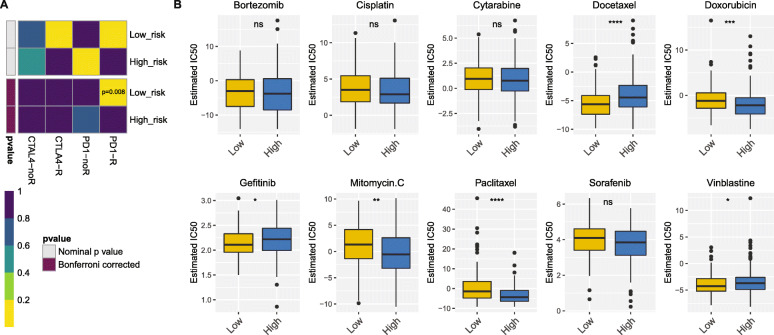
The prediction of immunotherapeutic and chemotherapeutic responses. **A**) SubMap analysis revealed that the low-risk group was more sensitive to PD-1 inhibitor (Bonferroni-corrected *P* = 0.008); **B**) The predicted IC50 for chemotherapeutic drugs in the low- and high-risk group. The low-risk group was related to a lower IC50 in docetaxel, gefitinib and vinblastine, while the high-risk group was related to a lower IC50 in doxorubicin, mitomycin C and paclitaxel (*P* < 0.05 by Wilcoxon test)

Since more TILs significantly enriched in the low-risk group patients, we attempted to further investigate whether the immunotherapies response are different in the low- and high-risk group. TIDE prediction suggest that there was no significantly difference in immunotherapies response between the low- (48.61%, 70/144) and high-risk (42.58%, 66/155) group (*P* = 0.297). However, by mapping the expression profile of the low- and high-risk group with a public dataset of 47 melanoma patients responded to immunotherapies in SubMap modules of GenePattern [60], the low-risk group showed prospective response to anti-PD-1 (programmed cell death protein 1) therapy (Bonferroni-corrected *P* = 0.008, Fig. 8A). Besides immunotherapies, we attempted to identify whether the 25-lncRNA prognostic signature could be applied to chemotherapies prediction. The results showed that the low-risk group had a lower half maximal inhibitory concentration of docetaxel, gefitinib and vinblastine, while the high-risk group had a lower half maximal inhibitory concentration of doxorubicin, mitomycin C and paclitaxel (Fig. 8B, *P* < 0.05). Thus, the 25-lncRNA prognostic signature could act as a potential predictor for immunotherapies and chemotherapies.

## Discussion

As a class of non-coding transcripts, lncRNAs have been identified in all model organisms. So far, over 56,000 human lncRNAs have been reported in recent lncRNA annotations and the number of lncRNAs keeps growing [61]. Unlike protein-coding genes, most lncRNAs are less conserved, which leads to neglect of the function of lncRNAs [62]. However, accumulating evidence mainly at cellular level has indicated the involvement of lncRNAs in various biological processes such cell proliferation, apoptosis and nutrient sensing to cell differentiation [63]. Moreover, dysregulation of lncRNAs has been implicated in the pathogenesis of various diseases including cancers [64]. Many lncRNAs have shown their prognostic value in many types of cancers [26, 27]. In this study, we established a 25-lncRNA risk signature to predict HCC early recurrence. We demonstrated that, compared to AFP, TNM and VI, this 25-lncRNA risk signature possesses the best prognostic potential for HCC early recurrence. Moreover, the combination of the lncRNA risk signature with AFP, TNM and VI could further improve the predictive performance.

In this study, we define the recurrence in 2-year post-surgery as HCC early recurrence. This is in agreement with a previous study showing that the slopes of early recurrence curve and late recurrence curve are different and the intercept time point of the two curves is defined as the cut-off to separate early and late recurrence [4]. This separation criterion is widely adopted by many other studies [65]. We also noticed that some studies define the recurrence in 1-year post-surgery as HCC early recurrence [66, 67]. Therefore, we analyzed the association of the 25-lncRNA risk signature with both 1-year and 2-year recurrence in most of our analyses and found that this risk signature has great prognostic potential for both of them.

The 25-lncRNA risk signature includes 19 risk lncRNAs (coefficient > 0) and 6 protective lncRNAs (coefficient < 0) (Table 1). Among these lncRNAs, dysregulation of LINC02159, CLDN10-AS1, LOC643201, LRP4-AS1, LOC730100, LINC01697, LOC100505622, and LINC00261 has been reported in several types of cancers (Table 1). In addition, CLDN10-AS1 was reported to be involved in endothelial dysfunction in atherogenesis (Table 1). Some previous studies have suggested the association of LOC153910 with lung function development, risk of chronic obstructive pulmonary disease (COPD) and cardiovascular diseases (CVD) (Table 1). LINC00261 has shown to regulate endoderm differentiation, lung epithelial homeostasis and endometriosis (Table 1). Given the fact that most of lncRNAs demonstrate a tissue-specific expression pattern [68], further investigation of the role of those 25 lncRNAs in HCCs is warranted.

To investigate the biological processes or pathways related to HCC early recurrence, we performed GSEA to explore the hallmarks of gene sets in the high-risk group. Total 8 gene sets were significantly enriched in the high-risk group. Among them, the gene sets of “E2F TARGETS”, “G2M CHECKPOINT”, “MYC TARGETS V1” and “DNA REPAIR” showed higher significance in enrichment. In fact, members of those four gene sets have been reported to associate with poor prognosis in many types of cancers including HCC [69–72].

Accumulation of TILs is commonly related to an improved prognosis in many types of cancers. In the present study, greater intratumor accumulation of TILs was observed in the low-risk group compared to high-risk group. We demonstrated that the 25-signature risk score significantly and negatively associate with intratumor accumulation of type 1 T helper cell, effector memory CD8 T cell and activated CD8 T cell, which are well-known antitumor immune cells involved in cancer immune therapy [73–75], further suggesting that this 25-lncRNA signature has potential to predict the post-surgery prognosis in HCC patients. In addition, the immunotherapies prediction based on this 25-lncRNA signature suggested that the low-risk group had more effective response to PD-1 inhibitor. Moreover, chemotherapies prediction indicated that the low- and high-risk showed different sensitivity to drugs such as docetaxel and paclitaxel, but not cisplatin and sorafenib. Thus, different therapies might be adapted to HCC patient in the low- and high risk group according to the 25-lncRNA signature.

Although the 25-lncRNA risk signature was validated in the TCGA internal validation cohort and displayed good prognostic potential in the enrolled 299 HCC patients, an external validation cohort is missing in this study. This is because we failed to find any suitable GEO datasets or International Cancer Genome Consortium (ICGC) database which could apply sufficient information on both lncRNA expression profile and clinical survival. For example, there are two GEO datasets, GSE67260 and GSE113850, possess satisfied data on lncRNA expression profile but without clinical records. Moreover, expression profiles of 22 lncRNAs in the 25-lncRNA signature could be extracted from two ICGC datasets including LIRI-JP and LICA-FR, but disease free survival information is missing. The Cancer Genome Atlas (TCGA) is a multi-institutional, cross-discipline effort led by the National Cancer Institute recruiting cancer samples from different countries. For example, the HCC samples recruited in the TCGA-LIHC database were derived from Vietnam, United States, Canada, South Korea, Russia [76]. Therefore, those samples are actually derived from multi-centers and at certain level support the approach we used in this study by splitting them into a training cohort and a validation cohort. However, validation of this 25-lncRNA risk signature in an external cohort will be warranted as long as suitable data are available. Meanwhile, the validation of individual lncRNA included in this 25-lncRNA signature in clinical HCC tumor and paracancerous tissues were in processing but have not completed yet. LncRNAs ENSG00000231918, ENSG00000248596, and ENSG00000223392 were found to be upregulated in 36 HCC tumor tissues compared with paracancerous tissues (Fig. S7, Table S4).

## Conclusions

In this study, we established a 25-lncRNA risk signature for HCC early recurrence. According to this risk signature, HCC patients could be accurately separated into the low- and high-risk groups. 1-year and 2-year recurrence rates were significantly higher in the high-risk group than those in the low-risk group. More importantly, the risk score significantly and negatively correlates with DFS in recurrent HCC patients in the high-risk group. Univariate and multivariate Cox regression analyses showed that the 25-lncRNA risk score, serum AFP, TNM stage and vascular invasion (VI) were independent prognostic factors for HCC early recurrence. Moreover, compared to serum AFP, TNM stage and VI, the 25-lncRNA risk signature showed better prognostic potential for HCC early recurrence. In addition, the combination of the 25-lncRNA risk signature with serum AFP, TNM stage and VI could further improve the prognostic potential for HCC early recurrence. Meanwhile, GSEA showed that several gene sets related to malignancy, such as “E2F TARGETS”, “G2M CHECKPOINT”, “MYC TARGETS V1” and “DNA REPAIR”, have significantly enriched in the high-risk group, suggesting that lncRNAs included in this risk signature may affect HCC progression through those biological pathways. Moreover, ssGSEA revealed greater TILs in the low-risk group compared to the high-risk group and the negative association between the 25-lncRNA risk signature score and the intratumor population of several key antitumor TILs such as type 1 T helper cell, effector memory CD8 T cell and activated CD8 T cell, and SubMap algorithm predicted that the low-risk group was more sensitive to anti-PD-1 therapy. Finally, Chemotherapies prediction revealed that the low risk was associated with sensitivity to docetaxel, gefitinib and vinblastine, while high risk was associated with sensitivity to doxorubicin, mitomycin C and paclitaxel.

## Supplementary Information

**Additional file 1.**

## Data Availability

The dataset supporting the conclusions of this article is available in the TCGA-LIHC repository, http://cancergenome.nih.gov/.

## Abbreviations

HCC: Hepatocellular carcinoma
lncRNA: long non-coding RNA
TCGA: The Cancer Genome Atlas
DEG: Differentially expressed gene
LASSO: Least absolute shrinkage and selection operator
ROC: Receiver operating characteristic curve
GSEA: Gene set enrichment analysis
TIL*s*: Tumor infiltrating lymphocytes
ssGSEA: single sample Gene Set Enrichment Analysis
GDSC: Genomics of Drug Sensitivity in Cancer
AJCC: American Joint Committee on Cancer
BCLC: Barcelona Clinic Liver Cancer
CLIP: Cancer of the Liver Italian Program
AFP: Alpha-fetoprotein
FDA: Food and Drug Administration
CRC: Colorectal cancer
LIHC: Liver Hepatocellular Carcinoma
OS: Overall survival
DFS: Disease free survival
ER lncRNAs: lncRNAs associated with HCC early recurrence
VI: Vascular invasion
NES: Normalized enrichment scores
PD-1: Programmed cell death protein 1
COPD: Chronic obstructive pulmonary disease
CVD: Cardiovascular diseases
ICGC: International Cancer Genome Consortium
