# Supplementary material for: Identification and characterization of a 25-lncRNA prognostic signature for early recurrence in hepatocellular carcinoma
Source: BMC Cancer. 2021 Oct 30;21:1165. doi: 10.1186/s12885-021-08827-z (PMC8556945; doi:10.1186/s12885-021-08827-z)
Supplement: Supplementary file 1 — Additional file 1. [file 12885_2021_8827_MOESM1_ESM.pdf]

Supplementary information for

Identification and characterization of a 25-lncRNA prognostic signature for early recurrence in hepatocellular carcinoma

Yi Fu[1,2,3], Xindong Wei[4], Qiuqin Han[1,2], Jiamei Le[1,2], Yujie Ma[2], Xinjie Lin[2], Yuhui Xu[5], Ning Liu[6], Xuan Wang[7], Xiaoni Kong[8], Jinyang Gu[9*], Ying Tong[10*] and Hailong Wu[1.2*]

[1]Affiliated Zhoupu Hospital, Shanghai University of Medicine and Health Sciences, Shanghai, 201318, China

[2]Shanghai Key Laboratory of Molecular Imaging, Collaborative Innovation Center for Biomedicines, Shanghai University of Medicine and Health Sciences, Shanghai, 201318, China

[3]School of Medical Instruments, Shanghai University of Medicine and Health Sciences, Shanghai, 201318, China

[4]Nanjing University of Traditional Chinese Medicine, Nanjing, 210000, China

[5]Graduate School of Art and Sciences, Columbia University, New York, NY 10027, United States

[6]Department of Clinical Oncology, Taian City Central Hospital, Taian, Shandong 271000, China

[7]Department of General Surgery, Nanjing General Hospital of Nanjing Military Command, Nanjing, 210000, China

[8]Institute of Clinical Immunology, Department of Liver Diseases, Central Laboratory, ShuGuang Hospital Affiliated to Shanghai University of Traditional Chinese Medicine, Shanghai 200021, China

[9]Department of Transplantation, Xinhua Hospital Affiliated to Shanghai Jiao Tong University School of Medicine, Shanghai, 200092, China

[10]Department of Liver Surgery, Renji Hospital Affiliated to Shanghai Jiao Tong University School of Medicine, Shanghai, 200127, China

[*]Corresponding Author:
Prof. Hailong Wu,
email: wuhl@sumhs.edu.cn
Dr. Ying ~~Dong~~,
email: lilytongy@hotmail.com
Dr. Jinyang Gu,
email: gjynyd@126.com

SUPPLEMENTARY FIGURES and FIGURE LEGENDS

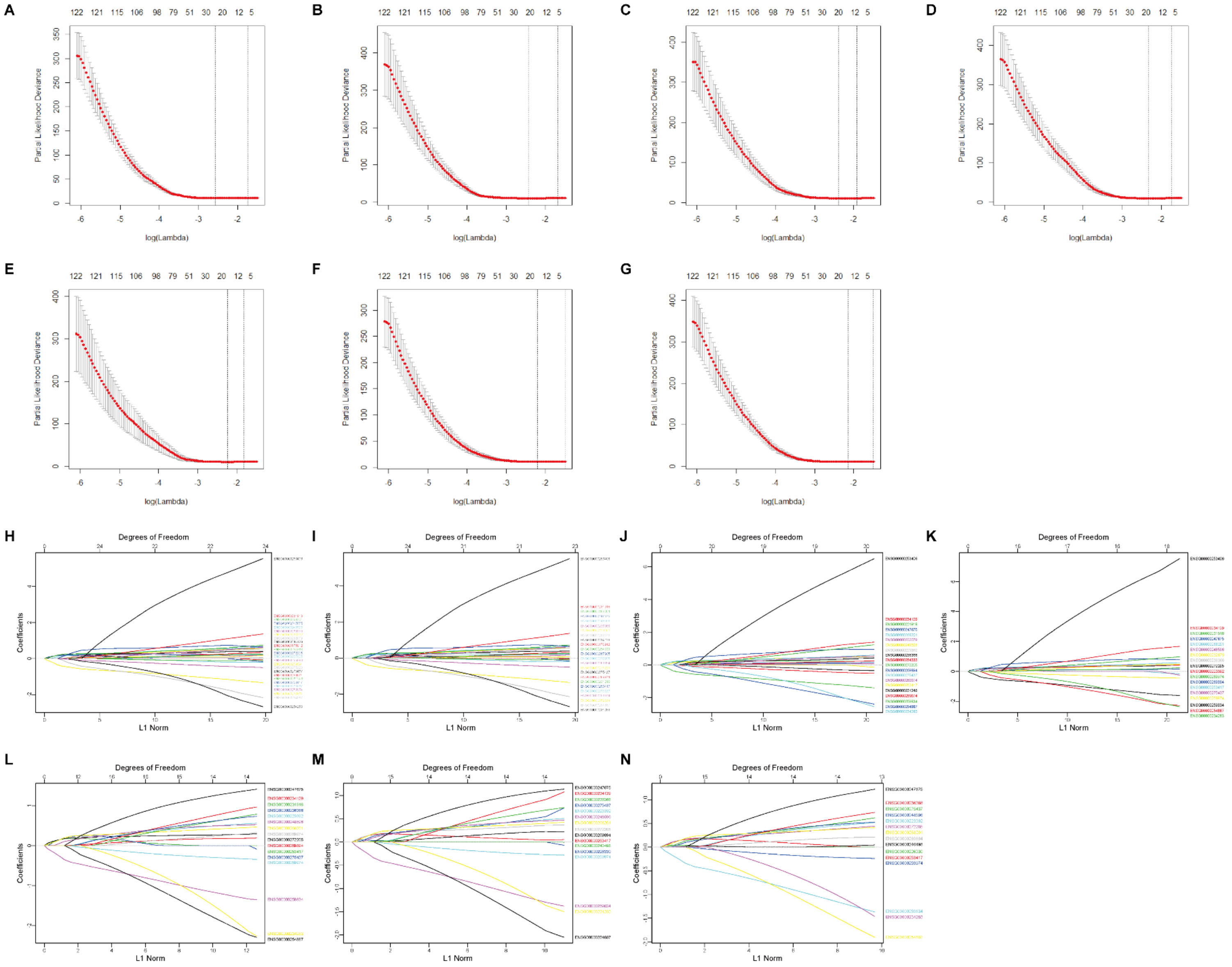

Figure S1 LASSO regression results of 7 constructed LncRNA-based risk signature

A-G) Ten-fold cross-validation for tuning parameter selection in the LASSO model. The dotted vertical lines are drawn at the optimal values by minimum criteria (lambda.min, left vertical dotted line) and 1-SE criteria (lambda.1se, right vertical dotted line); lambda.min is 0.07681466, 0.0883182, 0.09252353, 0.09692911, 0.1063796, 0.1114449 and 0.1167515 in A)-G) respectively; H-N) Partial LASSO coefficient profiles of the 358 lncRNAs. The dotted vertical line was plotted at the value selected using 10-fold cross-validation in A)-G), for which the optimal λ (lambda.min) resulted in H) 25 non-zero coefficients for A); I) 24 non-zero coefficients for B); J) 20 non-zero coefficients for C); K) 18 non-zero coefficients for D); L) 16 non-zero coefficients for E); M) 16 non-zero coefficients for F); N) 15 non-zero coefficients for G)

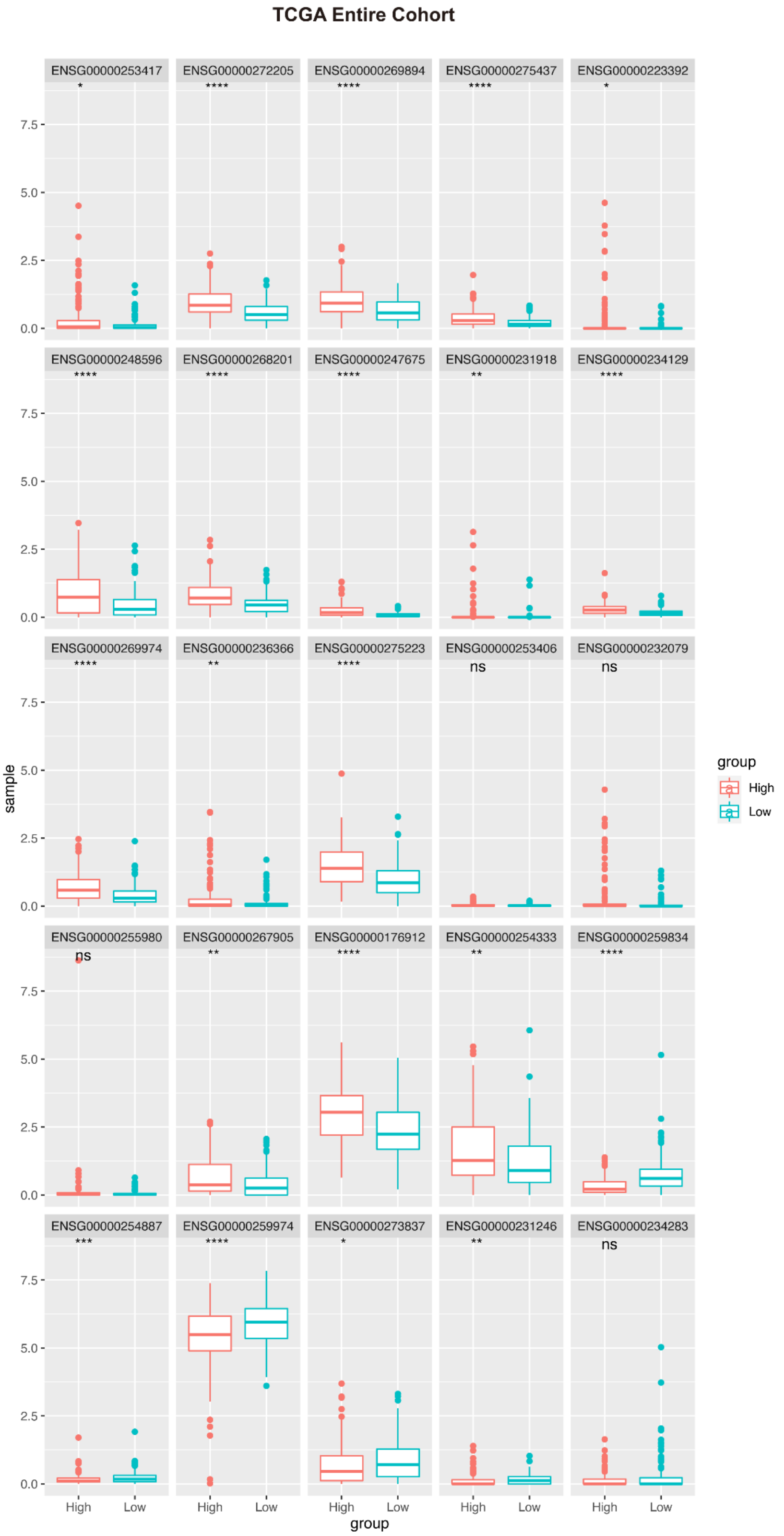

Figure S2 The expression comparison of lncRNAs in the low- and high-risk groups

Non-pair Wilcoxon test was used to compared the expression of lncRNAs between low- and high groups (ns: non-specific; *: P<0.05; **: P<0.01; ***: P<0.001, ****: P<0.0001).

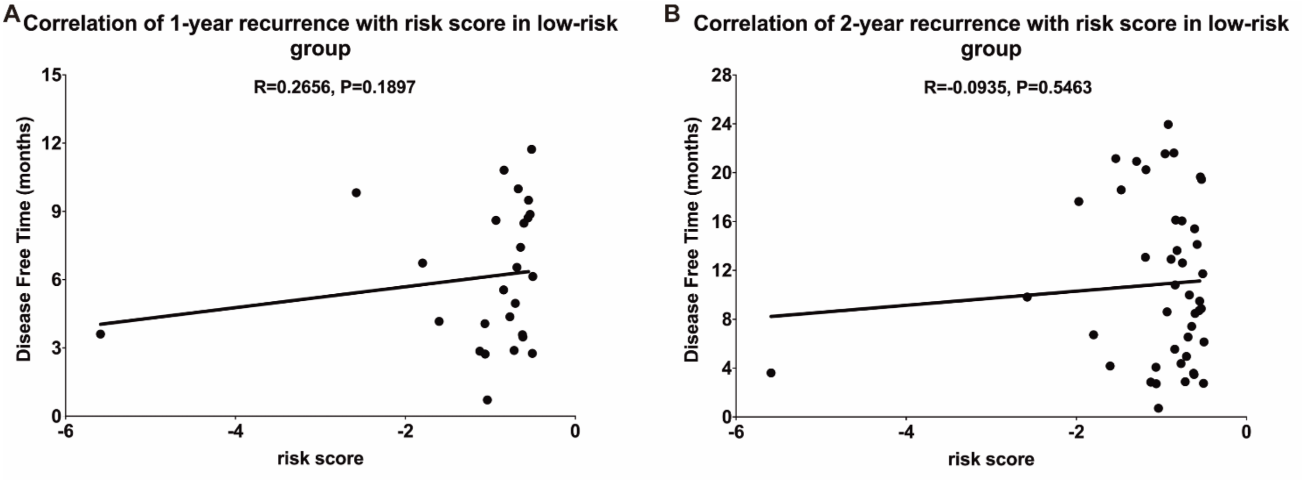

Figure S3 Correlation analysis of recurrence with risk score in the low-risk group

A-B) Correlation between risk score and 1-year DFS (R=0.2656, P=0.1897) (A) and 2-year DFS (R=-0.0935, P=0.5463) (B).

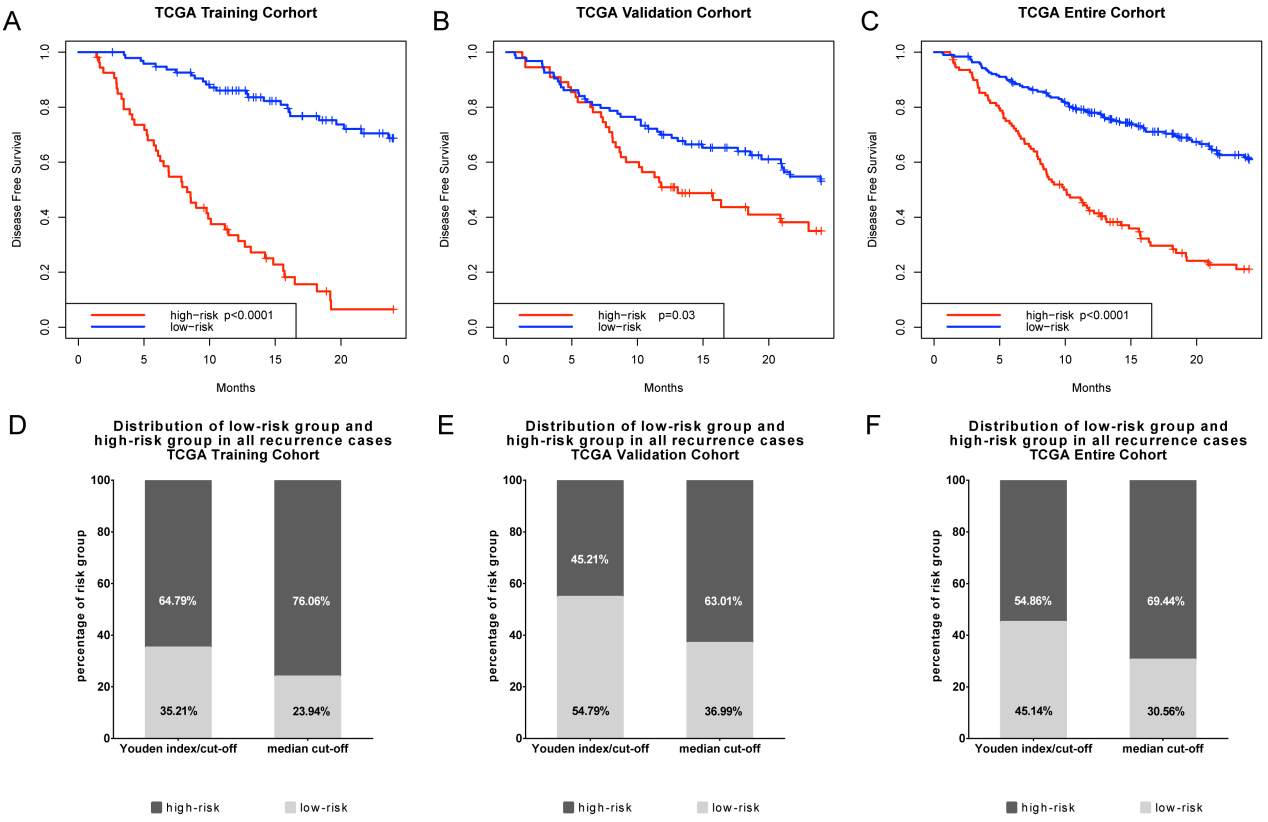

Figure S4 Comparison of different cut-off threshold

A)-C) KM plot of the 25-lncRNA risk signature with 2-year DFS was analyzed in the training cohort (N=150, P<0.0001) (A), validation cohort (N=149, P=0.03) (B), and the entire TCGA cohort (N=299, P<0.0001) (C). The statistical significance was determined by the log-rank test. The patients in each cohort were stratified into the high-risk and low risk groups based on the cut-off risk score set to -0.2 in the training cohort. D) 64.79% (cut-off=-0.2) and 76.06% (cut-off=median) HCC patients with recurrence were classified in the high-risk group based on different cut-off, and 35.21% (cut-off=-0.2) and 23.94% (cut-off=median) HCC patients with recurrence were assigned in the low-risk group; E) 45.21% (cut-off=-0.2) and 63.01% (cut-off=median) HCC patients with recurrence were classified in the high-risk group based on different cut-off, and 54.79% (cut-off=-0.2) and 36.99% (cut-off=median) HCC patients with recurrence were assigned in the low-risk group; F) 54.86% (cut-off=-0.2) and 69.44% (cut-off=median) HCC patients with recurrence were classified in the high-risk group based on different cut-off, and 45.14% (cut-off=-0.2) and 30.56% (cut-off=median) HCC patients with recurrence were assigned in the low-risk group.

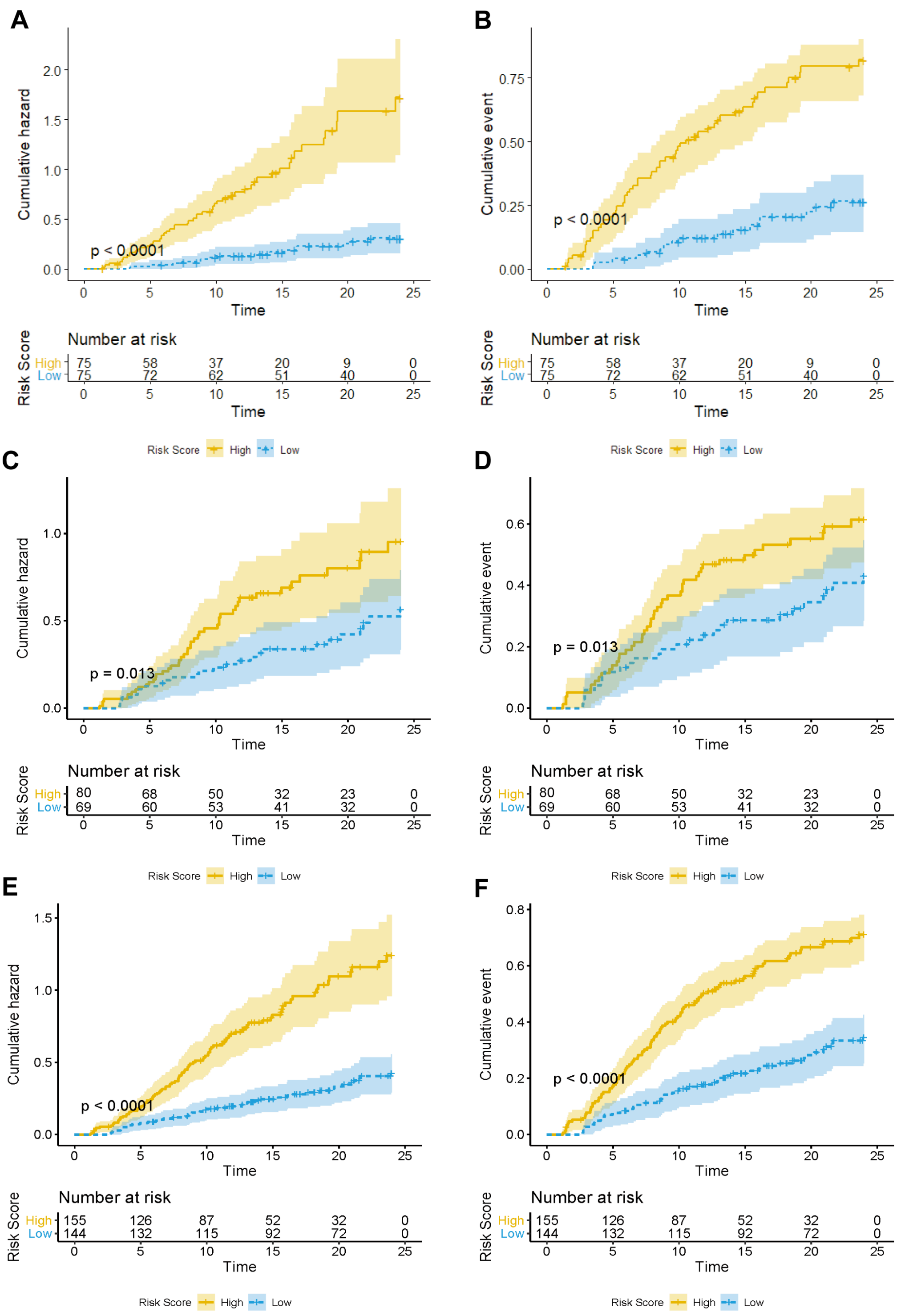

Figure S5 Recurrence analysis of HCC patients

A, C, E) Cumulative hazard curves showed higher recurrence hazard in the high-risk group of the training cohort (N=150) (P<0.0001) (A), validation cohort (N=149) (P=0.015) (C) and entire TCGA cohort (N=299) (P<0.0001) (E); B, D, F) Cumulative event curves showed higher recurrence events in the high-risk group of the training cohort (N=150) (P<0.0001) (B), validation cohort (N=149) (P=0.015) (D) and entire TCGA cohort (N=299) (P<0.0001) (F).

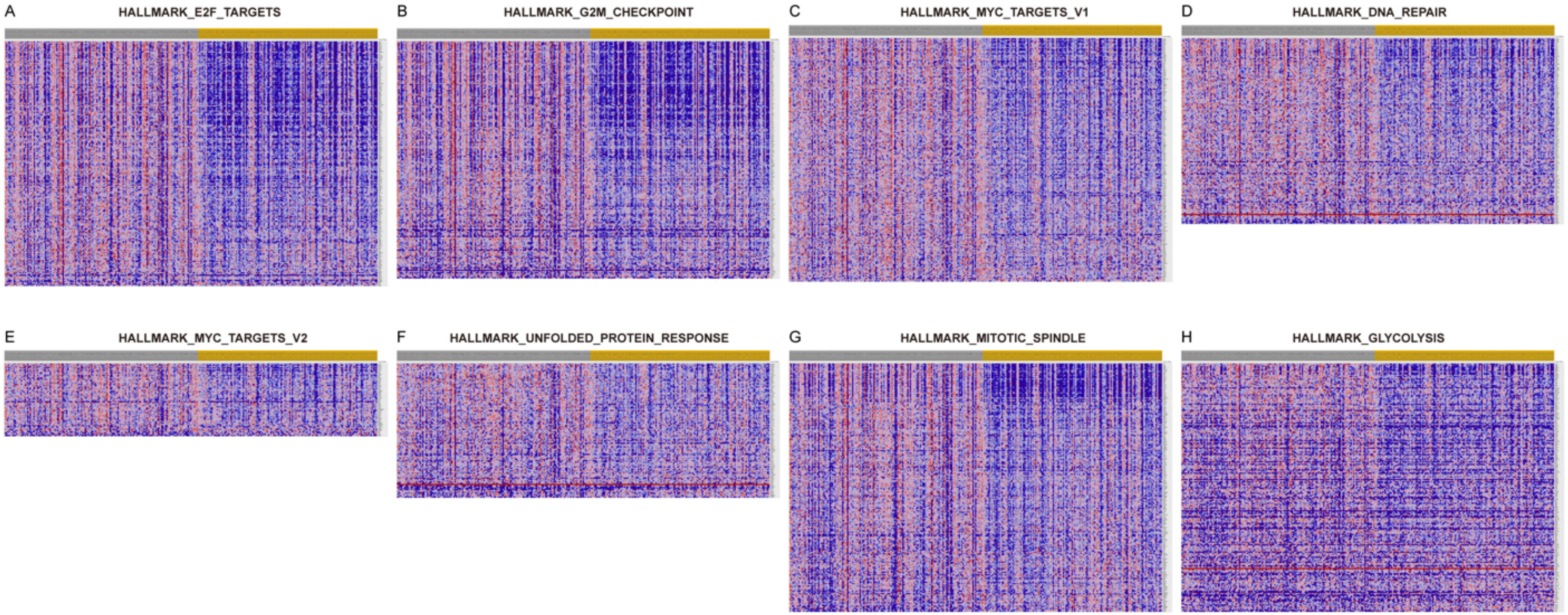

Figure S6 Heatmap for the genes of the significant pathways

Heatmap of genes in the low-risk group (brown) and the high-risk group (grey) of A) HALLMARK_E2F_TARGETS; B) HALLMARK_G2M_CHECKPOINT; C) HALLMARK_MYC_TARGETS_V1; D) HALLMARK_DNA_REPAIR; E) HALLMARK_MYC_TARGETS_V2; F) HALLMARK_UNFOLDED_PROTEIN_RESPONSE; G) HALLMARK_MITOTIC_SPINDLE; H) HALLMARK_GLYCOLYSIS.

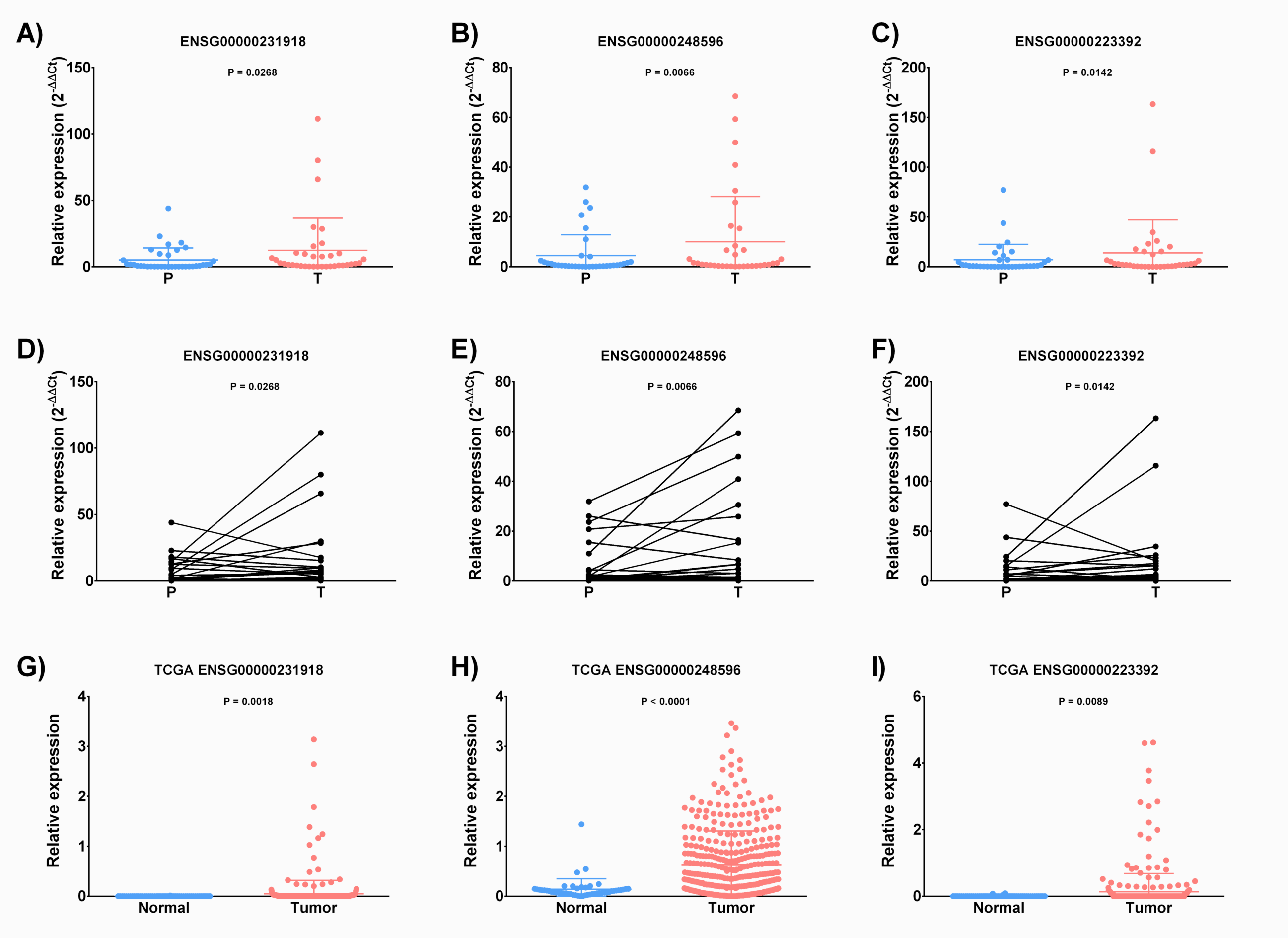

Figure S7 LncRNA expression in clinical samples and TCGA database

A)-C) Relative expressions of lncRNAs were higher in clinical HCC tissues than those in paracancerous tissues (n=36) for A) ENSG00000231918 (P = 0.0268), B) ENSG00000248596 (P = 0.0066), and C) ENSG00000223392 (P = 0.0142); D)-E) 36 Paired HCC tissues and paracancerous tissues ;G)-I) Relative expressions of lncRNAs were higher in TCGA HCC samples (N=373) than those in normal samples (N=50) for G) ENSG00000231918 (P = 0.0018), H) ENSG00000248596 (P < 0.0001), and I) ENSG00000223392 (P = 0.0089).

SUPPLEMENTARY TABLES

Table S1 Clinical characteristics of 299 HCC patients involved in the study

Characteristics	Number of Cases
**Disease Free Survival (299/299)**	
Disease Free	125(41.81%)
Recurred/Progressed	174(58.19%)
**Overall Survival (299/299)**	
Alive	220(73.58%)
Dead	79(26.42%)
**TNM Stage (281/299)**	
Stage I	145(51.6%)
Stage II	67(23.84%)
Stage III+IV	69(24.56%)
**Fibrosis (181/299)**	
Established Cirrhosis	62(34.25%)
Fibrous Speta	22(12.15%)
No Fibrosis	60(33.15%)
Nodular Formation and Incomplete Cirrhosis	7(3.87%)
Portal Fibrosis	30(16.57%)
**Vascular Invasion (251/299)**	
Macro	13(5.18%)
Micro	73(29.08%)
None	165(65.73%)
**AFP Range (236/299)**	
≤ 20 ng/ml	133(56.36%)
>20 ng/ml	103(43.64%)
**Race (292/299)**	
AMERICAN INDIAN OR ALASKA NATIVE	1(0.34%)
ASIAN	133(45.55%)
BLACK OR AFRICAN AMERICAN	11(3.77%)
WHITE	147(50.34%)
**Sex (299/299)**	
Female	90(30.1%)
Male	209(69.9%)
**Age Range (299/299)**	
≤ 50	60(20.07%)
>50	239(79.93%)

Table S2 7 group of lncRNAs sequence summarized from LASSO regression analysis

Model	NO. of LncRNAs	LncRNAs
1	25	ENSG00000253417, ENSG00000272205, ENSG00000269894, ENSG00000275437, ENSG00000223392, ENSG00000248596, ENSG00000268201, ENSG00000247675, ENSG00000231918, ENSG00000259834, ENSG00000234129, ENSG00000269974, ENSG00000254887, ENSG00000259974, ENSG00000236366, ENSG00000275223, ENSG00000253406, ENSG00000232079, ENSG00000273837, ENSG00000255980, ENSG00000267905, ENSG00000176912, ENSG00000254333, ENSG00000231246, ENSG00000234283
2	24	ENSG00000253417, ENSG00000272205, ENSG00000269894, ENSG00000275437, ENSG00000223392, ENSG00000248596, ENSG00000268201, ENSG00000247675, ENSG00000231918, ENSG00000259834, ENSG00000234129, ENSG00000269974, ENSG00000254887, ENSG00000259974, ENSG00000236366, ENSG00000275223, ENSG00000253406, ENSG00000232079, ENSG00000273837, ENSG00000267905, ENSG00000176912, ENSG00000254333, ENSG00000231246, ENSG00000234283
3	20	ENSG00000253417, ENSG00000272205, ENSG00000269894, ENSG00000275437, ENSG00000223392, ENSG00000248596, ENSG00000268201, ENSG00000247675, ENSG00000231918, ENSG00000259834, ENSG00000234129, ENSG00000269974, ENSG00000254887, ENSG00000259974, ENSG00000236366, ENSG00000253406, ENSG00000232079, ENSG00000254333, ENSG00000231246, ENSG00000234283
4	18	ENSG00000253417, ENSG00000272205, ENSG00000269894, ENSG00000275437, ENSG00000223392, ENSG00000248596, ENSG00000268201, ENSG00000247675, ENSG00000231918, ENSG00000259834, ENSG00000234129, ENSG00000269974, ENSG00000254887, ENSG00000259974, ENSG00000236366, ENSG00000253406, ENSG00000232079, ENSG00000234283
5	16	ENSG00000253417, ENSG00000272205, ENSG00000269894, ENSG00000226330, ENSG00000240498, ENSG00000275437, ENSG00000223392, ENSG00000248596, ENSG00000268201, ENSG00000247675, ENSG00000259834, ENSG00000234129, ENSG00000254887, ENSG00000259974, ENSG00000236366, ENSG00000234283
6	16	ENSG00000253417, ENSG00000272205, ENSG00000269894, ENSG00000275437, ENSG00000223392, ENSG00000248596, ENSG00000268201, ENSG00000247675, ENSG00000231918, ENSG00000259834, ENSG00000234129, ENSG00000269974, ENSG00000254887, ENSG00000259974, ENSG00000236366, ENSG00000234283
7	15	ENSG00000253417, ENSG00000272205, ENSG00000269894, ENSG00000226330, ENSG00000240498, ENSG00000275437, ENSG00000223392, ENSG00000248596, ENSG00000268201, ENSG00000247675, ENSG00000259834, ENSG00000254887, ENSG00000259974, ENSG00000236366, ENSG00000234283

Table S3 AUC and C-index of predication models

	1-year DFS			2-year DFS		
	AUC	95% CI	C-index	AUC	95% CI	C-index
RS+TNM+VI+AFP	78.8%	72.02%-85.56%	0.75	76.8%	70.3%-83.35%	0.74
RS	73.9%	66.49%-81.22%	0.71	72.0%	64.95%-79.02%	0.70
TNM	65.0%	57.63%-72.36%	0.63	67.2%	60.52%-73.83%	0.63
VI	63.5%	56.4%-70.55%	0.62	60.3%	53.9%-66.76%	0.60
AFP	64.6%	57.56%-71.6%	0.63	61.4%	54.73%-68.06%	0.61

Table S4 Clinical information of 36 HCC patients

Patient NO	Gender	Age	HBsAg	HCVAb	Cirrhosis	AFP	ALT	Tumor Size	Tumor Number	TNM	Recurrence
1	Female	50	+	-	+	1028	55	12	1	III	Not Available
2	Female	71	-	-	-	18.4	12	8	1	I	Not Available
3	Male	61	-	+	-	3.02	19	1.8	1	I	Not Available
4	Female	64	+	-	+	8.67	61	5.5	>1	III	Not Available
5	Male	64	+	-	+	4.75	14	6	1	I	Not Available
6	Male	76	-	-	-	1.21	17	4	1	I	Not Available
7	Female	35	+	-	-	1185	20	15	1	III	Not Available
8	Female	64	-	-	-	3.65	24	4.5	1	I	Not Available
9	Male	65	+	-	-	8256	47	9	1	I	Not Available
10	Male	67	+	-	-	4.84	18	4.5	1	II	Not Available
11	Male	60	+	-	+	2.46	22	2	1	I	Not Available
12	Male	62	+	-	-	81.7	32	2	1	III	Recurrence
13	Female	55	+	-	-	3.08	27	9.2	1	III	Not Available
14	Male	60	+	-	-	2.38	42	4.5	1	I	Not Available
15	Male	56	+	-	+	1438	32	1.5	1	I	Not Available
16	Male	75	-	-	-	8.35	28	8	1	I	Not Available
17	Male	47	+	-	-	39241	26	4	>1	III	Not Available
18	Male	59	+	-	+	2.29	12	1.5	>1	II	Not Available
19	Male	69	+	-	+	2884	26	3	>1	III	Recurrence

20	Male	55	+	-	-	14.7	23	17	>1	III	Recurrence
21	Male	54	+	-	-	40.6	36	4.5	1	I	Not Available
22	Male	64	+	-	-	2.47	17	3	>1	II	Not Available
23	Female	66	+	-	+	423	56	9	>1	III	Recurrence
24	Female	71	-	-	-	611	8	6	1	III	Not Available
25	Male	47	+	-	+	>60500	62	20	1	IV	Recurrence
27	Male	43	+	-	+	2.27	27	1.3	>1	II	Not Available
28	Female	66	+	-	+	5.1	20	3	1	I	Not Available
29	Male	67	-	-	+	5832	33	2.8	>1	IV	Recurrence
30	Male	60	+	-	+	550.1	33	6	1	IV	Recurrence
33	Female	53	-	+	+	2.63	35	3.7	1	I	Not Available
34	Female	47	+	-	-	2.01	26	16	>1	III	Not Available
35	Male	29	+	-	+	24.7	83	9	>1	III	Recurrence
36	Male	62	+	-	-	205	21	5.5	1	I	Not Available
37	Female	50	+	-	+	11.3	72	3	>1	II	Not Available
38	Male	41	+	-	+	2072	31	12	>1	III	Recurrence
40	Male	54	+	-	-	1241	80	14	>1	III	Not Available

SUPPLEMENTARY REFERENCE in Table 1

1. Nasir, S. N., Abu, N., Ab Mutalib, N. S., Ishak, M., Sagap, I., Mazlan, L., Rose, I. M., and Jamal, R. (2018) LOC285629 regulates cell proliferation and motility in colorectal cancer cells. *Clin Transl Oncol* **20**, 775-784
2. Sun, L., Guan, Z., Wei, S., Tan, R., Li, P., and Yan, L. (2019) Identification of Long Non-coding and Messenger RNAs Differentially Expressed Between Primary and Metastatic Melanoma. *Front Genet* **10**, 292
3. Zhu, Z., Wang, X., Li, X., Lin, Y., Shen, S., Liu, C. L., Hobbs, B. D., Hasegawa, K., Liang, L., Boezen, H. M., Camargo, C. A., Jr., Cho, M. H., and Christiani, D. C. (2019) Genetic overlap of chronic obstructive pulmonary disease and cardiovascular disease-related traits: a large-scale genome-wide cross-trait analysis. *Respir Res* **20**, 64
4. Jin, D., Song, Y., Chen, Y., and Zhang, P. (2020) Identification of Three lncRNAs as Potential Predictive Biomarkers of Lung Adenocarcinoma. *Biomed Res Int* **2020**, 7573689
5. Fan, Q., and Liu, B. (2018) Comprehensive analysis of a long noncoding RNA-associated competing endogenous RNA network in colorectal cancer. *Onco Targets Ther* **11**, 2453-2466
6. Huang, Y. L., Ning, G., Chen, L. B., Lian, Y. F., Gu, Y. R., Wang, J. L., Chen, D. M., Wei, H., and Huang, Y. H. (2019) Promising diagnostic and prognostic value of E2Fs in human hepatocellular carcinoma. *Cancer Manag Res* **11**, 1725-1740
7. Zhang, Y., Jin, T., Shen, H., Yan, J., Guan, M., and Jin, X. (2019) Identification of Long Non-Coding RNA Expression Profiles and Co-Expression Genes in Thyroid Carcinoma Based on The Cancer Genome Atlas (TCGA) Database. *Med Sci Monit* **25**, 9752-9769
8. Li, Q., Lu, J., Xia, J., Wen, M., and Wang, C. (2019) Long non-coding RNA LOC730100 enhances proliferation and invasion of glioma cells through competitively sponging miR-760 from FOXA1 mRNA. *Biochem Biophys Res Commun* **512**, 558-563
9. Liu, J., Yao, Y., Hu, Z., Zhou, H., and Zhong, M. (2019) Transcriptional profiling of long-intergenic noncoding RNAs in lung squamous cell carcinoma and its value in diagnosis and prognosis. *Mol Genet Genomic Med* **7**, e994
10. Wang, X., Wan, J., Xu, Z., Jiang, S., Ji, L., Liu, Y., Zhai, S., and Cui, R. (2019) Identification of competitive endogenous RNAs network in breast cancer. *Cancer Med* **8**, 2392-2403
11. Li, X. X., Wang, L. J., Hou, J., Liu, H. Y., Wang, R., Wang, C., and Xie, W. H. (2020) Identification of Long Noncoding RNAs as Predictors of Survival in Triple-Negative Breast Cancer Based on Network Analysis. *Biomed Res Int* **2020**, 8970340
12. Shiozaki, A., Ariyoshi, Y., Iitaka, D., Kosuga, T., Shimizu, H., Kudou, M., Konishi, T., Shoda, K., Arita, T., Konishi, H., Komatsu, S., Kubota, T., Fujiwara, H., Okamoto, K., Kishimoto, M., Konishi, E., Marunaka, Y., Ichikawa, D., and Otsuji, E. (2019) Functional analysis and clinical significance of sodium iodide symporter expression in gastric cancer. *Gastric Cancer* **22**, 473-485
13. Yu, Y., Li, L., Zheng, Z., Chen, S., Chen, E., and Hu, Y. (2017) Long non-coding RNA linc00261 suppresses gastric cancer progression via promoting Slug degradation. *J Cell Mol Med* **21**, 955-967
14. Zhang, H. F., Li, W., and Han, Y. D. (2018) LINC00261 suppresses cell proliferation, invasion and Notch signaling pathway in hepatocellular carcinoma. *Cancer Biomark* **21**, 575-582

15. Wang, Z. K., Yang, L., Wu, L. L., Mao, H., Zhou, Y. H., Zhang, P. F., and Dai, G. H. (2017) Long non-coding RNA LINC00261 sensitizes human colon cancer cells to cisplatin therapy. *Braz J Med Biol Res* **51**, e6793
16. Wang, Y., Xue, K., Guan, Y., Jin, Y., Liu, S., Wang, L., and Han, L. (2017) Long Noncoding RNA LINC00261 Suppresses Cell Proliferation and Invasion and Promotes Cell Apoptosis in Human Choriocarcinoma. *Oncol Res* **25**, 733-742
17. Shahabi, S., Kumaran, V., Castillo, J., Cong, Z., Nandagopal, G., Mullen, D. J., Alvarado, A., Correa, M. R., Saizan, A., Goel, R., Bhat, A., Lynch, S. K., Zhou, B., Borok, Z., and Marconett, C. N. (2019) LINC00261 Is an Epigenetically Regulated Tumor Suppressor Essential for Activation of the DNA Damage Response. *Cancer Res* **79**, 3050-3062
18. Lin, K., Jiang, H., Zhuang, S. S., Qin, Y. S., Qiu, G. D., She, Y. Q., Zheng, J. T., Chen, C., Fang, L., and Zhang, S. Y. (2019) Long noncoding RNA LINC00261 induces chemosensitization to 5-fluorouracil by mediating methylation-dependent repression of DPYD in human esophageal cancer. *FASEB J* **33**, 1972-1988
19. Liu, Y., Xiao, N., and Xu, S. F. (2017) Decreased expression of long non-coding RNA LINC00261 is a prognostic marker for patients with non-small cell lung cancer: a preliminary study. *Eur Rev Med Pharmacol Sci* **21**, 5691-5695
20. Singh, K. K., Matkar, P. N., Pan, Y., Quan, A., Gupta, V., Teoh, H., Al-Omran, M., and Verma, S. (2017) Endothelial long non-coding RNAs regulated by oxidized LDL. *Mol Cell Biochem* **431**, 139-149
21. Kreiner-Moller, E., Bisgaard, H., and Bonnelykke, K. (2014) Prenatal and postnatal genetic influence on lung function development. *J Allergy Clin Immunol* **134**, 1036-1042 e1015
22. Soler Artigas, M., Wain, L. V., Miller, S., Kheirallah, A. K., Huffman, J. E., Ntalla, I., Shrine, N., Obeidat, M., Trochet, H., McArdle, W. L., Alves, A. C., Hui, J., Zhao, J. H., Joshi, P. K., Teumer, A., Albrecht, E., Imboden, M., Rawal, R., Lopez, L. M., Marten, J., Enroth, S., Surakka, I., Polasek, O., Lyytikainen, L. P., Granell, R., Hysi, P. G., Flexeder, C., Mahajan, A., Beilby, J., Bosse, Y., Brandsma, C. A., Campbell, H., Gieger, C., Glaser, S., Gonzalez, J. R., Grallert, H., Hammond, C. J., Harris, S. E., Hartikainen, A. L., Heliovaara, M., Henderson, J., Hocking, L., Horikoshi, M., Hutri-Kahonen, N., Ingelsson, E., Johansson, A., Kemp, J. P., Kolcic, I., Kumar, A., Lind, L., Melen, E., Musk, A. W., Navarro, P., Nickle, D. C., Padmanabhan, S., Raitakari, O. T., Ried, J. S., Ripatti, S., Schulz, H., Scott, R. A., Sin, D. D., Starr, J. M., Vinuela, A., Volzke, H., Wild, S. H., Wright, A. F., Zemunik, T., Jarvis, D. L., Spector, T. D., Evans, D. M., Lehtimaki, T., Vitart, V., Kahonen, M., Gyllensten, U., Rudan, I., Deary, I. J., Karrasch, S., Probst-Hensch, N. M., Heinrich, J., Stubbe, B., Wilson, J. F., Wareham, N. J., James, A. L., Morris, A. P., Jarvelin, M. R., Hayward, C., Sayers, I., Strachan, D. P., Hall, I. P., and Tobin, M. D. (2015) Sixteen new lung function signals identified through 1000 Genomes Project reference panel imputation. *Nat Commun* **6**, 8658
23. Shagiwal, S. S., den Dekker, H. T., de Jongste, J. C., Brusselle, G. G., Jaddoe, V. W. V., Felix, J. F., and Duijts, L. (2018) Influence of genetic variants on childhood lung function - The Generation R Study. *Pediatr Allergy Immunol* **29**, 589-595
24. Castaldi, P. J., Cho, M. H., Litonjua, A. A., Bakke, P., Gulsvik, A., Lomas, D. A., Anderson, W., Beaty, T. H., Hokanson, J. E., Crapo, J. D., Laird, N., and Silverman, E. K. (2011) The association of genome-wide significant spirometric loci with chronic obstructive pulmonary disease susceptibility. *Am J Respir Cell Mol Biol* **45**, 1147-1153

25. Jiang, W., Liu, Y., Liu, R., Zhang, K., and Zhang, Y. (2015) The lncRNA DEANR1 facilitates human endoderm differentiation by activating FOXA2 expression. *Cell Rep* **11**, 137-148
26. Swarr, D. T., Herriges, M., Li, S., Morley, M., Fernandes, S., Sridharan, A., Zhou, S., Garcia, B. A., Stewart, K., and Morrisey, E. E. (2019) The long noncoding RNA Falcor regulates Foxa2 expression to maintain lung epithelial homeostasis and promote regeneration. *Genes Dev* **33**, 656-668
27. Sha, L., Huang, L., Luo, X., Bao, J., Gao, L., Pan, Q., Guo, M., Zheng, F., and Wang, H. (2017) Long non-coding RNA LINC00261 inhibits cell growth and migration in endometriosis. *J Obstet Gynaecol Res* **43**, 1563-1569
